# New Adenosine Derivatives from *Aizoon canariense* L.: In Vitro Anticholinesterase, Antimicrobial, and Cytotoxic Evaluation of Its Extracts

**DOI:** 10.3390/molecules26051198

**Published:** 2021-02-24

**Authors:** Riham O. Bakr, Mohammed F. El-Behairy, Ahmed M. Elissawy, Hanan Elimam, Marwa A. A. Fayed

**Affiliations:** 1 Department of Pharmacognosy, Faculty of Pharmacy, October University for Modern Sciences and Arts (MSA), Giza 11787, Egypt; romar@msa.eun.eg; 2 Department of Organic and Medicinal Chemistry, Faculty of Pharmacy, University of Sadat City, Sadat 32897, Egypt; mohammed.farrag@fop.usc.edu.eg; 3 Department of Pharmacognosy, Faculty of Pharmacy, Ain Shams University, Cairo 11566, Egypt; aelissawy@pharma.asu.edu.eg; 4 Center for Drug Discovery Research and Development, Ain Shams University, Cairo 11566, Egypt; 5 Department of Biochemistry, Faculty of Pharmacy, University of Sadat City, Sadat 32897, Egypt; Hanan.Elimam@fop.usc.edu.eg; 6 Department of Pharmacognosy, Faculty of Pharmacy, University of Sadat City, Sadat 32897, Egypt

**Keywords:** *Aizoon canariense*, Aizoaceae, adenosine alkaloids, anticholinesterase, cytotoxicity, antimicrobial

## Abstract

Aizoaceae is a large succulent family characterized by many psychoactive species. *Aizoon canariense* L., a wild neglected plant traditionally used in gastrointestinal ailments, has been the subject of a limited number of phytochemical and biological studies. Therefore, herein, we investigated the in vitro cytotoxic, antimicrobial, and anticholinesteraseactivity of the aerial parts of *A. canariense* L. and analyzed the phytochemical compositions of the lipoidal and alkaloidal fractions. Petroleum ether extract showed the presence of behenic and tricosylic acid, while an in-depth investigation of the alkaloidal fraction revealed the identification of new adenine based alkaloids (1–5), which were isolated and identified for the first time from *Aizoon canariense* L. Their structures were elucidated based on extensive spectroscopic analyses. The alkaloidal extract showed a powerful cytotoxic effect (IC_50_ 14–28 μg/mL), with the best effect against colon carcinoma, followed by liver and breast carcinomas. The alkaloidal extract also had a potent effect against *Candida albicans* and *Escherichia coli*, with minimum inhibitory concentrations (MIC) values of 312.5 and 625 µg/mL. The in vitro anticholinesterase activity was potent, with IC_50_ < 200 ng/mL for the tested extracts compared with 27.29 ± 0.49 ng/mL for tacrine.

## 1. Introduction

African plants have been proven to have encouraging healing powers but are scarce in scientific investigations. Many studies have shown promising antimicrobial, cytotoxic, and enzyme inhibitory effects, correlated with the diversity of their phytoconstituents [1,2]

Aizoaceae, the “ice plant”, is the largest family of succulent leaves, consisting of 135 genera and 2499 species. Aizoaceae is characterized by its flavonoids, as reported in *Aptenia* [3,4] and *Trianthema* [5,6], and alkaloids, as identified in *Sceletium* species [7], in addition to sterols and triterpenes [8]. Alkaloids of the family Aizoaceae are characterized by their phenolic alkaloid nature [7,9]. Six genera and ten species are recognized from Aizoaceae in Egypt, however are poorly studied [10]. *Aizoon* is one of the wildly grown genera of the subfamily Aizooideae [11]. *Aizoon canariense* L. (Gafna or Shafna) was traditionally used to treat gastrointestinal problems and as a hypotensive [12]. Limited studies have dealt with its chemical profile, demonstrating the presence of simple phenolics besides flavonoids such as quercetin [13], fatty acids such as lauric acid, and stearidonic acid, as well as omega 3 [14]. A recent study allowed the identification of flavonoid glycosides, sterols, and adenosine [15]. *A. canariense* L. was also reported to have moderate antioxidant and antibacterial activities [12,16], in addition to cytotoxic activity, against liver carcinoma [15]. 

Cancer is the second leading cause of death worldwide, causing around 9.6 million deaths in 2018. Although a huge number of research studies have investigated new drugs and treatment regimens, this number continues to increase, with 300,000 new cases diagnosed annually [17]. Plants can still apply their magic through a range of natural products, with alkaloids representing a very promising class, with antiproliferative and anti-angiogenic effects [18]. Researchers have also given attention to infectious diseases, being one of the top ten causes of death globally, mainly due to antimicrobial resistance and the decline in the number of new antibiotics being discovered. The interest in natural products as anti-infective agents dates back to before the discovery of penicillin by Alexander Fleming, from the usage of molds and herbs to treat infections [19,20]. Alkaloids are major weapons in this war [21]. 

Besides these activities, alkaloids have been reported to have beneficial effects in reducing the deteriorative effects of Alzheimer’s, acting with an anticholinesterase-like activity through their different classes [22]. In this study, different extracts of *A. canariense* L. were investigated for their biological activities, including cytotoxic, antimicrobial, anticholinesterase-related activities, whereby the alkaloidal fraction was the target for the isolation and identification of constituents using 1D and 2D NMR spectral techniques. We also report the isolation of adenine-based alkaloids for the first time in *A. canariense* L.

## 2. Results and Discussion

### 2.1. Chemical Characterization

**Figure 1:**
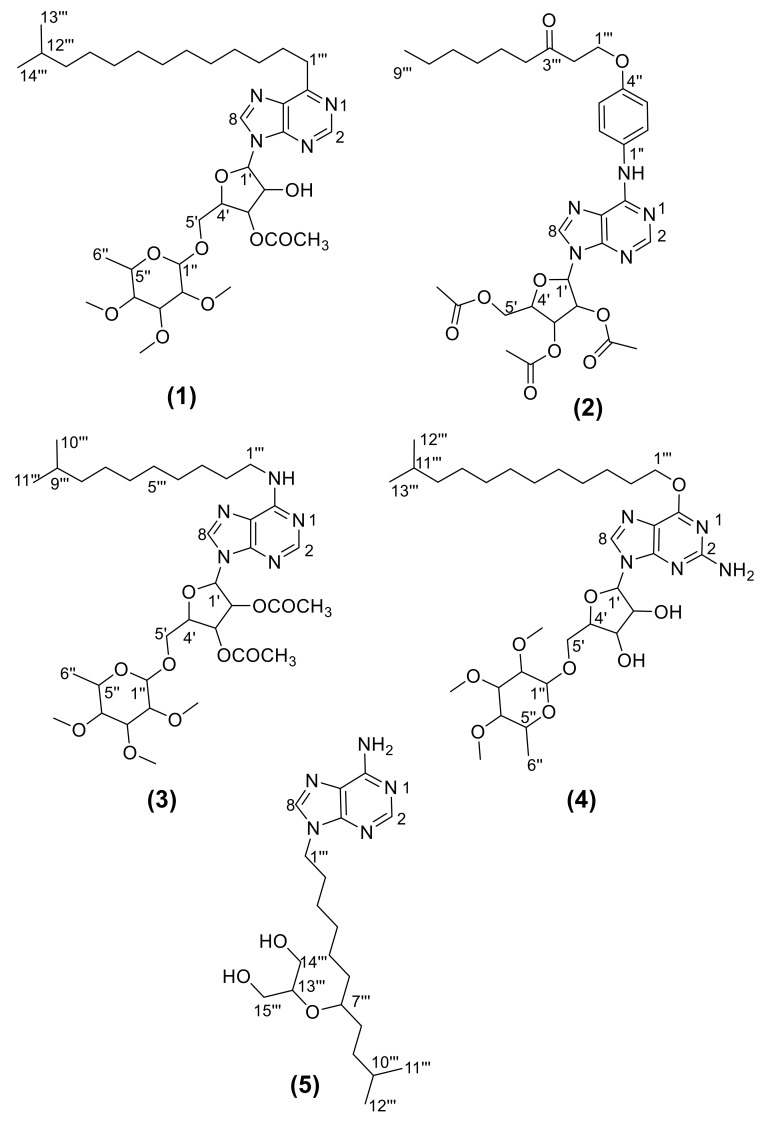
Structures of compounds **1**–**5** isolated from aerial parts of *A. canariense* L.

Purine bases and nucleosides are produced by the turnover of nucleotides and nucleic acids, as well as from some cellular metabolic pathways [23]. Purine base is not limited to the xanthine alkaloids but it encompasses adenine and guanine glycosides [24]. Combined information from 1D and 2D NMR (COSY, and HSQC) experiments were utilized to predict the structures of compounds **1**–**5** (Figure 1, Figures S2–S16). The 1D ^1^H-NMR spectra of compound **2** showed the presence of 3 protons corresponding to H-2, H-8, and NH protons at δ_H_ 8.76, 8.58, and 5.67 ppm, respectively, characteristic of alkaloids with an adenine nucleus [25]. A set of 4 aromatic protons was detected at δ_H_ 7.23–7.42 ppm corresponding to H-2′′, 3′′, 5′′, and 6′′. H-1′′′ was noticed as a singlet at δ_H_ 3.65 ppm, while H-5′′′–8′′′ were observed as a broad signal corresponding to 8Hs at δ_H_ 1.29 ppm. H9′′′ was observed at δ_H_ 1.21–1.23 ppm as doublet signal counting for 3Hs. H-2′′′ and 4′′′ were displayed as two singlet signals at δ_H_ 2.88 and 2.11 ppm, respectively, and directly after the broad signal corresponding to acetate methyl groups of ribose at δ_H_ 2.05 ppm and counting for 9Hs. Ribose protons were observed at δ 3.0–3.6 ppm. The later values were in agreement with the reported data by Ciuffreda et al. for adenine nucleoside acetates [25]. The signals arising due to anomeric protons are usually reported to appear in the range of 4.0–5.9 ppm, while the protons of α-glycosides typically resonate 0.3–0.5 ppm downfield from those of the corresponding β-glycosides [26]. In compound **2**, the anomeric H1′ appeared as a doublet signal at δ_H_ 4.03–4.05 ppm (*J* = 8.0 Hz), which is in the range of β-glycosides. Additionally, the coupling constant between H-1′ and H-2′ is 8.0 Hz. Ciuffreda et al. introduced further confirmation of anomeric configuration via the evaluation of differences between δ-values of H-2 and H-1′. The magnitude of these differences is larger than 2.15 ppm in β-derivatives and smaller than 1.85 ppm in the α-ones [25]. In our case, the difference between δ-values of the H-2 and H-1′ was 4.72, which is more than 2.15, so it should be a β-anomer. 

The 1D APT ^13^C-NMR of compound **2** demonstrated the sugar moiety methines at δ_c_ 70–83 ppm and were phased negatively, while C-2′′′, 4′′′, and 1′′′ methylenes were reported at δ_c_ 50.52, 52.38, and 59.62 ppm, respectively, and were phased positively. Further methylenes (C-5′′′–9′′′) were reported at δ_c_ 25–32 ppm. The methyl at C-10′′′ was observed at δ_c_ 20.82 ppm and further methyls of acetates were detected at δ_c_ 25.39 and 31.69 ppm and were phased negatively. The C-3′′′ carbonyl was noticed at δ_c_ 206 ppm and was phased positively, while acetate carbonyls were observed in HSQC (Figure S5). Ester carbonyls appeared at δ_c_ 174–177 and showed coupling with both sugar and methyl Hs (Table 1). Additionally, the coupling of sugar Cs and Hs of adjacent carbons was spotted in HSQC. The characteristic coupling of ketone carbonyl C-3′′′, H-4′′′, and H-2′′′ was noticed in HSQC (207.51, 2.11 and 207.29, 2.88 ppm, respectively). In the HSQC, 6 couplings in the aromatic region were noticed that corresponded to H-2, 8, 2′′, 3′′, 5′′, and 6′′. A characteristic coupling between C-2′′′ and H-2′′′ was observed at δ_H_ 2.88, 50.45 ppm. Additionally, couplings of ribose carbons and protons were noticed. The positive MS spectrum revealed an [M]^+^ peak at *m/z* 625, suggesting a molecular formula of C_31_H_39_N5O_9_; therefore, compound **2** was identified as *N*-(4′′-((3′′′-oxononyl)oxy)phenyl)-β-adenosine-2′,3′,5′-triacetate, isolated for the first time from aerial parts of *A. canariense* L.

An additional adenine derivative was characterized (compound **3**), where signals of H-2, H-8, and NH protons were detected at δ_H_ 8.50, 8.42, and 7.8 ppm, respectively. Compound **3**—in contrast to compound **2**—showed no additional aromatic protons, however methine protons of acetylated ribose were observed, as follows: H1′ at δ_H_ 4.56–4.59 ppm as a triplet signal *J* = 10.68 Hz (β-anomer) and H2′-H5′ at δ_H_ 3.48–3.51 ppm, while acetate methyls of ribose were noticed at δ_H_ 2.48–2.59 ppm. Surprisingly, compound **3** was recognized as a disaccharide nucleoside [27]. The second sugar moiety was identified as fully methylated rhamnopyranoside. Even though the methylated rhamnopyranoside is not common in nature, it has been previously reported by Sone et al. [28]. The chemical shifts of the methylated rhamnopyranoside moiety of compound **3** were in the same range as that previously reported by Sone et al. Thus, 3′′, 4′′, and 5′′-OCH_3_ were detected at δ_H_ 3.48–3.51 ppm as a singlet signal for 9Hs. Additionally, rhamnopyranose methine proton H1′′ appeared at δ_H_ 4.56–4.59 (t, *J* = 5.12 Hz, *α*-anomer), while H2′′–H5′′ were noticed at δ_H_ 3.41–3.51 ppm. The methyl group at C-6′′ was detected at δ_H_ 1.23 ppm. The alkyl side chain protons were detected at δ_H_ 1.21–1.24 (H3′′′–H8′′′), 1.72–1.73 (H2′′′ and H9′′′) ppm, and 0.84 ppm (H10′′′ and 11′′′) (Figures S7–S10). ^13^C-NMR of compound **3** demonstrated the sugar moieties methines at δ_c_ 60–73 ppm. Methyls of acetates were detected at δ_c_ 20.29 and 21.56, while acetate carbonyls were detected at δ_c_ 177 ppm. Methyls at C10′′′ and C11′′′ were noticed at δ_c_ 14.37 ppm, Rhamnopyranose methyl 6′′ was detected at δ_c_ 17.87 ppm and 3′′, 4′′ and 5′′-OCH_3_ were detected at δ_c_ 70 ppm. C1′′′ methylene was reported at δ_c_ 66 ppm, while C2′′′-9′′′ methylenes were detected at δ_c_ 20–30 ppm. In HSQC, the signal at δ_H_ 0.84 was linked to δ_c_ 14.37 ppm (terminal alkyl methyls at C10′′′ and C11′′′). Rhamnopyranose methyl 6′′ was detected at δ_H_ 1.23 and was related to δ_c_ 17.87 ppm. Several couplings corresponding to the alkyl side chain methylenes were detectable at δ_H_ 1.21, 1.24, 1.72, and 1.73 and correlated to δ_c_ 25–30 ppm. Further couplings of sugar methines and rhamnopyranose methoxy groups were observed at δ_H_ 3.41–3.51 and δ_c_ 60–75 ppm. Characteristic couplings of anomeric atoms were picked out at δ_H_ 4.5–4.6 and δ_c_ 70 ppm. The positive high-resolution electrospray ionization mass spectrometry (HR-ESI-MS) spectrum (Figure S1) revealed an [M + H]^+^ peak at *m/z* 694.6000, suggesting a molecular formula of C_34_H_55_N_5_O_10_, which was as identified as *N*-(9′′′-methyldecyl)-β-adenosine-2′,3′-diacetate-2′′,3′′,4′′-tri-*O*-methyl-α-rhamnopyranoside.

Compound **4** was recognized as the non-acetylated guanosine nucleoside analogue of compound **3**. Thus, only one proton was detectable in the aromatic region at δ_H_ 7.70 ppm that corresponds to H-8, while no signals corresponding to H-2 were detectable. Additionally, NH_2_ protons were detectable at δ_H_ 5.32 ppm. H1′ of ribose was demonstrated at δ_H_ 4.11–4.14 ppm as a triplet signal *J* = 9.88 Hz (β-anomer). Further ribose protons were noticed at δ_H_ 3.34–3.57 ppm. Similar shifts of methylated rhamnopyranoside in compound **3** were demonstrated. Thus, H1′′′ detected at δ_H_ 4.11–4.14 ppm (*t*, 3.8 Hz, α-anomer) and H2′′-H5′′ was elucidated at δ_H_ 3.34–3.57 ppm, while H6′′ at δ_H_ 1.23 ppm and 3′′, 4′′, 5′′-OCH_3_ was detected at δ_H_ 3.51 ppm as a singlet signal for 9Hs. The alkyl side chain was formed of 13 carbons. H3′′′–H11′′′ were detected at δ_H_ 1.21–1.24 ppm, H2′′′ at δ 2.00 ppm, while H1′′′ appeared at δ_H_ 3.34–3.45 ppm and H12′′′–H13′′′ at δ_H_ 0.85 ppm (Figures S11–S13). ^13^C-NMR of compound **4** revealed the sugar moiety methines at δ_c_ 60–73 ppm. Methyls at C12′′′ and C13′′′ were noticed at δ_c_ 11 ppm, rhamnopyranose methyl 6′′ at δ_c_ 14 ppm, and 3′′–5′′-OCH_3_ at δ_c_ 70 ppm. C1′′′ methylene was observed at δ_c_ 67 ppm, while C2′′′–11′′′ methylenes were noticed at δ_c_ 22–33 ppm. The HSQC showed the correlation between H at δ_H_ 0.85 ppm and C at δ_c_ 11.0 and 14.0 ppm. Additionally, Hs at δ_H_ 1.21–1.24 ppm correlated with Cs at δ_c_ 22–33 ppm. Protons at δ_H_ 1.23 and 2.0 ppm were related to C at δ_c_ 25 and 27 ppm, respectively. Couplings of Hs at δ_H_ 3.34–3.57 ppm were discerned with carbons in the range of δ_c_ 60–75 ppm. The anomeric protons at δ_H_ 4.11–4.14 ppm were coupled with Cs at δ_c_ 65 and 70 ppm. The positive HR-ESI-MS spectrum (Figure S1) revealed an [M + H]^+^ peak at *m/z* 654.7000, suggesting a molecular formula of C_32_H_55_N_5_O_9_ identified as *O*-(11′′′-methyldodecyl)-β-guanosine-2′′,3′′,4′′-tri-*O*-methyl-*α*-rhamnopyranoside.

For compound **5**, the positive MS spectrum revealed an [M + H]^+^ peak at *m/z* 394, while the negative MS spectrum revealed an [M − H]^−^ peak at *m/z* 392, suggesting a molecular formula of C_20_H_35_N_5_O_3_ (Figure S1). Moreover, 1D ^1^H-NMR spectra showed typical adenine signals of H-2, H-8, and NH protons at δ_H_ 8.50, 7.97, and 5.44 ppm respectively. Neither aromatic nor sugar protons were detectable. Protons of methyl groups (H-11′′′ and 12′′′) were noticed as multiplet signal at δ_H_ 0.83–0.89 ppm. H-10′′′ was detected at δ_H_ 1.84 ppm while H2′′′-6′′′, 8′′′ and 9′′′ were noticed at δ_H_ 1.16–1.28 ppm and corresponding to 14Hs. H-14′′′ and 15′′′ were identified downfield as doublet signal at δ_H_ 2.35–2.37 ppm counting for 4 Hs. The most deshielded protons were spotted at δ_H_ 2.47 ppm (H-7′′′ and 13′′′) and 3.17 ppm (H-1′′′). In HSQC, H at δ_H_ 8.50 was correlated to C at 143.07, multiple signals at δ_H_ 0.83–0.89 ppm was connected to Cs at δ_c_ 12.24, 15.64, 22.27, and 23.25 ppm. Protons at δ_H_ 1.16–1.28 ppm were coupled with Cs at δ_c_ 21.14–31.18 ppm. Signals at δ_H_ 1.84, 2.35–2.37, and 2.47 ppm were associated with Cs at δ_c_ 22.76, 30.53, and 21.30 ppm respectively (Figures S14–S16). Compound **5** was identified as 2-((11-(6-amino-9*H*-purin-9-yl)-2-methylundecan-5-yl)oxy)propane-1,3-diol.

Compound **1** was also recognized as a purine derivative. The characteristic two protons of purine in the aromatic region corresponding to H8 and H2 were detected at δ_H_ 7.67–7.73 ppm. Signals of monoacetylated ribose, methylated rhamnopyranoside, and the alkyl side chain were noticeable in the same range as in compounds B, C, and D, as per Table 1 and Figure S2. Anomeric H1′ and H1′′ in sugar moieties were demonstrated at δ_H_ 4.13–4.15 ppm (q, 3.52, 5.56 Hz, α-anomers). The positive HR-ESI-MS spectrum (Figure S1) revealed an [M + H]^+^ peak at *m/z* 679.5000, suggesting a molecular formula of C_35_H_58_N_4_O_9_. The correlation of H and C of acetylated ribose; methylated rhamnopyranoside; and the aliphatic side chain of compounds **2, 3, 4**, and **5** were demonstrated by HSQC, as per Figures S2–S16. Compound **1** was identified as 6-(12′′′-methyltridecyl)-9*H*-purin-9-yl-3′-monoacetate-α-ribofuranoyl-2′′,3′′,4′′-tri-*O*-methyl-α-rhamnopyranoside.

### 2.2. Determination of the Lipoidal Matter

Fatty acids have a vital role in maintaining the structural integrity of cellular membranes, as well as being a great energy source and being present in signaling molecules Studies showed that Alzheimer’s disease patients have reduced levels of polyunsaturated and monounsaturated fatty acids [29,30], therefore supplementation of such fatty acids may ameliorate cognitive functions. GLC analysis of *A. canariense* L. petroleum ether extract allowed the identification of 91.7% of its fatty acid content, characterized by being long (C14–C22) and very long-chain fatty acids (more than 22 carbons) (Table 2). Saturated fatty acids showed their predominance, with tricosanoic acid (C23) being the major identified saturated fatty acid (43%), followed by behenic acid (C22), which has a role in skincare as an emollient and is able to restore the skin’s natural oils and improve overall levels of hydration [31]. Nervonic acid (C-24:1 Δ15, cis-15-tetracosenoic acid) is the major unsaturated fatty acid. It is a monounsaturated analog of lignoceric acid, which is known to enhance brain function and prevent demyelination. Additionally, it was proven to ameliorate memory function and to improve the activity of γ-glutamate cysteine ligase in the cerebral cortex [32,33]. Eicosenoic acid (C-20), also called gondoic acid, is a monounsaturated omega 9 fatty acid, which was reported to cause a mild reduction in NO levels and to reduce LPS-induced increase in iNOS, therefore having a mild anti-inflammatory effect [34].

### 2.3. Biological Activities

#### 2.3.1. In Vitro Cytotoxic Activity

The different *A. canariense* L. extracts were screened for their cytotoxic, antimicrobial, and acetylcholinesterase inhibitory activities (Table 3). Cytotoxic activity was evaluated for alkaloidal as well as methanolic extracts of *A. canariense* L., against three cancer cell lines, namely breast carcinoma (MCF-7), hepatocellular carcinoma (HepG-2), and colon carcinoma (HCT-116), using sample concentrations ranging from 0 to 500 μg/mL. The results of the cytotoxic activity of *A. canariense* L. extracts revealed that the alkaloidal extract had the most powerful effect (IC_50_ 14–28 μg/mL), with the best effect against HCT-116, followed by HepG-2 then MCF-7. The methanolic extract showed comparable results with higher IC_50_ (Table 3). The powerful cytotoxic potential of *A. canariense* L. was previously demonstrated against human CCRF-CEM leukemia cells [35] and HepG2 [15]. 

#### 2.3.2. Antimicrobial Activity

The antimicrobial activity levels of both methanolic and alkaloidal extracts were evaluated using the disc diffusion method against Gram-positive, Gram-negative, and fungi compared with reference antimicrobial and antifungal agents. MIC values were estimated for the most sensitive micro-organisms (Table 4 and Table 5). Through this study, the alkaloidal extract showed high activity against *Candida albicans* and *Salmonella typhimurium* and moderate activity against *Bacillus subtilis,* while the methanolic extract showed promising antifungal activity against *Aspergillus flavus*, which was comparable to ketoconazole, as well as moderate activity against *Staphylococcus aureus* and *Escherichia coli.* Comparing the MIC values of the alkaloidal and the methanolic extracts of *A. canariense* L., the alkaloidal extract had better activity against *Candida albicans* and *Escherichia coli*, with MIC values of 312.5 and 625 µg/mL, respectively, while the methanolic extract showed better activity against *Staphylococcus aureus* and *Bacillus subtilis,* with MIC values of 625 µg/mL for both. The potent antifungal effect was supported by a previous report demonstrating a powerful effect against *A. fumigatus* [16].

#### 2.3.3. Anticholinesterase Activity

**Figure 2:**
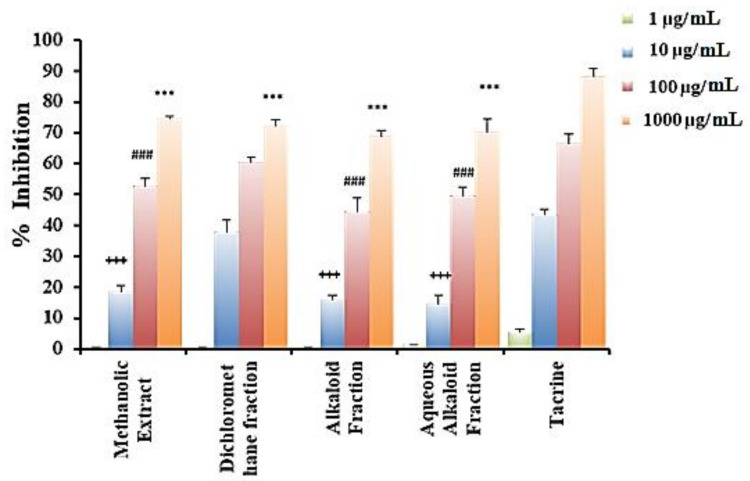
Percentage inhibition of the different *A. canariense* L. extracts. Note: *** *p* < 0.001 significant values compared to tacrine 1000 µg/mL; ^###^ *p* < 0.001 significant values compared to tacrine 100 µg/mL; ^+++^ *p* < 0.001 significant values compared to tacrine 10 µg/mL.

The increase in acetylcholinesterase (AChE) activity is the most characteristic change that occurs in Alzheimer’s disease. AChE is the enzyme responsible for acetylcholine hydrolysis, from both cholinergic and non-cholinergic neurons of the brain. The increase in acetylcholine level can be achieved by inhibition of AChE, which helps in the treatment of Alzheimer’s disease. The inhibitory activity of acetylcholinesterase was assessed using BioAssay Systems’ QuantiChromTM Screening kit based on an improved Ellman method. Through this work, the anti-Alzheimer’s activities (AChE enzyme inhibition activity) of the crude methanolic, dichloromethane, alkaloidal, as well as aqueous alkaloidal extracts of *A. canariense* L. were evaluated *in vitro* and compared with that of the standard tacrine (AChE inhibitor). The results are shown in Table 3 and Figure 2, representing the % inhibition levels at 10–1000 µg/mL and IC_50_ for the different extracts. The results demonstrated that the methanolic extract showed significant activity with IC_50_ = 112.24 ± 7.73 ng/mL, along with the aqueous alkaloid extract with IC_50_ =139.27 ± 21.40 ng/mL. Moreover, the dichloromethane extract showed the highest (very potent) and most promising anti-acetylcholinesterase activity with IC_50_ = 62.48 ± 1.31 ng/mL compared to the standard drug (AChE inhibitor) tacrine, with IC_50_ = 27.29 ± 0.49 ng/mL (Table 3). Taken together, these results demonstrated a considerable anti-Alzheimer’s activity of the extract. The ability of Aizoaceae plants to manage Alzheimer’s was previously demonstrated with *Trianthema portulacastrum*, mainly in the phenolic-rich fraction, where docking studies confirmed the significant binding affinity of chlorogenic acid towards AChE [36], while an in vivo model using *Sceletium tortuosum* showed cognitive set flexibility and executive function and positive changes in mood and sleep compared with the placebo group [37].

## 3. Experimental

### 3.1. Plant Material

Fresh whole-plant samples of *Aizoon canariense* L. (F. *Aizoaceae*) were collected from Cairo-Ismailia Road in February 2017 and kindly identified by Prof. Dr. A.A. Fayed, Professor of Plant Taxonomy, Faculty of Science, Assiut University, Assiut, Egypt. A voucher specimen is placed at the Herbarium of the Faculty of Science, Assiut University, Assiut, Egypt.

### 3.2. General Experimental Procedures

NMR analyses (^1^H, ^13^C, COSY, and HSQC) were performed on a Bruker instrument (Billerica, MA, USA; 400 and 100 MHz for ^1^H- and ^13^C-NMR, respectively) using DMSO-*d_6_* as a solvent and with chemical shift values given in δ (ppm) and referenced to the TMS signal as an internal reference. All samples were prepared in suitable deuterated solvents. An ultra-mass spectrometer was used (Thermo FisherScientific, Bremen, Germany), equipped with a Nanomate electrospray ionization (ESI) interface (Advion). An electrospray voltage of 1.7 kV (+/−) and a transfer capillary temperature of 200 °C were applied. Chromatographic analysis was carried out on TLC plates (Merck, Germany) using CH_2_Cl_2_–MeOH at different ratios, while column chromatographic separation was performed using a silica gel column with CH_2_Cl_2_ and gradient increase of MeOH. The analysis of fatty acid methyl esters was performed on an Agilent 19091J-413 gas chromatography instrument equipped with a flame ionization detector (FID), for which an HP-5 5% phenyl methyl siloxane capillary column (30 m × 320 µm × 0.25 µm) was used. The injector temperature was 250 °C, with an average velocity of 27 cm/s. H2 was the carrier gas, with a flow rate of 30 mL/min. The detector operated at a temperature of 280 °C. 

### 3.3. Extraction and Isolation

The dried and powdered plant material (0.5 kg) was defatted with petroleum ether until exhaustion, then plant material was exhaustively extracted with 95% methanol. Acid–base extraction was applied as previously reported [38], allowing the separation of the polar aqueous fraction (7 g) from the non-polar alkaloidal fraction (2 g), both giving a positive reaction with Dragendorff’s. The aqueous alkaloidal fraction (7 g) was chromatographed on a silica gel column (150 g, 100 × 3 cm) using a gradient elution of the CH_2_Cl_2_/MeOH mixture as a mobile phase. Then, 100 fractions were collected and similar fractions depending on TLC monitoring and visualization using Dragendorff’s reagent were gathered and concentrated, resulting in four main fractions. Fraction 1 (1–20) eluted with CH_2_Cl_2:_ MeOH (90:10) was neglected, as it consisted of traces of many compounds. Fraction 2 CH_2_Cl_2:_ MeOH (80:20) consisted of a mixture of 2 major spots, with some other impurities. Therefore, it was separated and purified using preparative high-performance liquid chromatography (HPLC) equipped with an RP-C18 column (Kromasil^®^, Bohus, Sweden; 5 µm, 250 mm × 10 mm), using a mobile phase consisting of 0.1% trifluroacetic acid in water (A)–MeOH (B) (HPLC grade) in the following sequence: 95% in 2 min, 95% to 50% in 23 min, 50% to 30% in 5 min. A photodiode array detector (Knauer K-2501, Berlin, Germany) was used. Samples were injected using a 100 µL glass syringe (VIGI syringe, USA; Knaur^®^ injector, D-14163, Berlin, Germany) yielding compounds **1** and **2** (10 and 20 mg, respectively). Fraction 3 (CH_2_Cl_2_: MeOH (70:30)) was further purified on another silica gel sub-column (60 g, 60 × 2 cm) using a gradient elution of the CH_2_Cl_2_–MeOH mixture as a mobile phase to yield compounds **3** (15 mg), **4** (20 mg), and **5** (10 mg). 

### 3.4. Determination of Lipoidal Matter

Here, 1 g of petroleum ether extract was refluxed with KOH for three hours, then the mixture was partitioned with diethyl ether. The aqueous layer was acidified with HCl, extracted with diethyl ether, and then the ethereal extract was esterified by refluxing with H_2_SO_4_–MeOH at a ratio of 3:50 for three hours. The ethereal layer was then collected and the residue was kept for GLC analysis. The identification of the fatty acid methyl esters was carried out by comparing retention times with the applied authentic sample. The quantitative estimation of each peak was achieved by using a computer integrator, adopting the internal normalization procedures [39]. 

### 3.5. Cell Culture

All cell lines used in this study were obtained from Nawah Scientific, Inc. (Mokatam, Cairo, Egypt). Cells were maintained in DMEM media supplemented with 100 mg/mL of streptomycin, 100 units/mL of penicillin, and 10% heat-inactivated fetal bovine serum in a humidified 5% (*v/v*) CO_2_ atmosphere at 37 °C.

### 3.6. Screening of Cytotoxic Activity

The cytotoxic activity of both methanolic and alkaloidal extracts was estimated using 3-(4,5-dimethylthiazol-2-yl)-2–5-diphenyltetrazolium bromide (MTT) assay against human breast cancer (MCF-7), liver cancer (HEPG2), and colon cancer (HCT-116) cell lines [40,41]. Principally, the MTT assay measures cell viability through the determination of the mitochondrial function of cells by measuring the activity of various mitochondrial enzymes (Stone V). Cell viability was determined using a cell proliferation kit [40] according to the manufacture’s protocol, while the optical density was measured at 590 nm with the microplate reader (SunRise, TECAN, Inc, Mannedorf, Switzerland) to determine the number of viable cells and the percentage of viability was calculated as (1 − (ODt/ODc)] × 100%), where ODt is the mean optical density of wells treated with the tested sample and ODc is the mean optical density of untreated cells. The relationships between surviving cells and drug concentrations were plotted to get the survival curve of each tumor cell line after treatment with the specified compound. The 50% inhibitory concentration (IC_50_), the concentration required to cause toxic effects in 50% of intact cells, was estimated from graphic plots of the dose–response curve for each concentration using GraphPad Prism v.8.4.2. (San Diego, CA, USA), as indicated. Here, *p* values < 0.05 were considered statistically significant.

### 3.7. Determination of Antimicrobial Activity

The minimum inhibitory concentration (MIC) values were determined for the different *A. canariense* L. extracts using the broth microdilution method against Gram-positive (*Staphylococcus aureus* (RCMB 010010), *Bacillus subtilis* (RCMB 015, NRRL B-543)) and Gram-negative bacteria (*Salmonella typhimurium* (RCMB 006, ATCC 14028), *Escherichia coli* (RCMB 010052, ATCC25955), in addition to fungi (*Aspergillus flavus* (RCMB 002002), *Candida albicans* (RCMB 005003, ATCC )), using 96-well microplates at the Regional Centre for Mycology and Biotechnology (RCMB) at AL-Azhar University, Nasr City, Egypt [42]. Microbial growth was indicated by the turbidity of the well. The lowest concentration showing no growth was taken as the minimum inhibitory concentration [43]. 

### 3.8. Anticholinesterase Activity

The most important enzyme controlling acetylcholine (ACh) levels in healthy brains is acetylcholinesterase (AChE), while butyrylcholinesterase (BChE) is involved to a lesser extent [44]. The anticholinesterase activity levels of the different *A. canariense* L. extracts were estimated using a QuantiChromTM kit, IACE-100, (BioAssay Systems, Hayward, CA, USA). The acetylcholinesterase inhibitor screening kit is dependent on an enzyme-catalyzed kinetic reaction [45]. The enzyme source, according to the manufacturer’s instructions, is *E. electricus*. The enzyme hydrolyzes the substrate acetylthiocholine, resulting in the production of thiocholine, which reacts with 5,5’-dithio-bis (2-nitrobenzoic acid) (DTNB) to form 2-nitrobenzoate-5-mercaptothiocholine and 5-thio-2-nitrobenzoate, which can be detected at 412 nm [46,47]. Briefly, and according to the manufacturer’s instructions, 45 μL samples of AChE (400 U/L) were incubated with 5 μL samples of tested extracts at a series of concentrations ranging from 1 to 500 μg/mL or 5 μL of 40 v% DMSO in a 96-well microplate. While in a separate well, 45 μL of assay buffer was used instead of AChE to achieve 100% inhibition in the negative control. The reaction mixture was incubated for 15 min at 37 °C. For each well, 150 μL of assay buffer was added, containing 1 μL substrate and 0.5 μL DTNB. The thiocholine produced by the action of acetylcholinesterase forms a yellow color with DTNB. The intensity of the produced color measured at 412 nm is proportionate to the enzyme activity in the sample. The optical density of the tested extracts was measured at 412 nm at 0 and 10 min in a plate reader compared with tacrine (Santa Cruz Biotechnology Cat# sc-200172) as standard (AChE inhibitor). The anticholinesterase activity was calculated as follows: % inhibition = 1 − (ΔOD _test_/ΔOD _control_) ×100 [48]. 

### 3.9. Statistical Analysis

Statistical analysis of the data was performed using one-way ANOVA, followed by Tukey’s multiple range test for post-hoc comparisons (GraphPad Prism, version 8.4.2). All the data are presented as the means of 3 determinations ± SE. The *p* value significance levels are represented as asterisks (*) for *p* < 0.05, (**) *p* < 0.01, and (***) *p* < 0.001.

## 4. Conclusions

This study provides the first report for the isolation and characterization of five adenine-based alkaloids from the polar alkaloidal fraction after acid–base extraction of the aerial parts of *A. canariense* L. The alkaloidal fraction of *A. canariense* L. showed a promising cytotoxic effect against HCT-116, MCF-7, and, HepG-2, in addition to significant antimicrobial effects. Furthermore, the alkaloid fraction, as well as dichloromethane (flavonoid containing fraction), showed a significant effect against Alzheimer’s disease, which requires further in vivo studies. The predominance of behenic and tricosanoic acids in the non-polar fraction, as well as the adenine-based alkaloids, may correlate to the potential effects in cerebral disorders. Our work revealed *A. canariense* L. a potential candidate for the treatment of many ailments.

## Figures and Tables

**Table 1:** ^1^H- and ^13^C-NMR data for compounds **1**–**5** (DMSO-*d*_6_).

Compound No.	Compound 1	Compound 2		Compound 3		Compound 4		Compound 5	
Position	δ_H_ [m, *J* (Hz)]	δ_H_ [m, *J* (Hz)]	δ_C_	δ_H_ [m, *J* (Hz)]	δ_C_	δ_H_ [m, *J* (Hz)]	δ_C_	δ_H_ [m, *J* (Hz)]	δ_C_
2	7.67–7.73	8.76 *s*	152	8.50	156	-	134	8.5 *s*	143
4	-	-	150	-	143	-	130	-	152
4a	-	-	-	-	-	-	-	-	-
5	-	-	121	-	140	-	128	-	120
6	-	-	156	-	157	-	134	-	157
8	7.67–7.73	8.58 *s*	140	8.42 (*d*, 5.28)	141	7.70 (*d*, 6.92)	129	7.97 *s*	140
NH_2_	-	5.67 *s*	-	7.8 (NH)	-	5.32	-	5.44 *s*	-
1′	4.13–4.15 (*q*, 3.52, 5.56, Hz)	4.01–4.05 (*t*, *J* = 8.0 Hz)	79	4.56–4.59 (*t*, 5.12, 10.68 Hz)	60–73	4.11–4.14 (*t*, 9.88 Hz)	60–73	-	-
2′	3.4–3.51	3.0–3.6		3.48–3.51		3.34–3.57		-	-
3′			73					-	-
4′			82					-	-
5′			62					-	-
2′,3′-OH	-	-	-	-	-	2.26	-	-	-
2′,3′-OCOCH_3_	3.4–3.51	2.05 *s*	174–177, 25.39–31.69	2.48–2.59	20.29–21.56– 177	-	-	-	-
1″	4.13–4.15 (*q*, 3.52, 5.56, Hz)			4.56–4.59 (*t*, 5.12 Hz, 10.68 Hz)	60–73	4.11–4.14 (*t*, 3.8 Hz)	60–73	-	-
2″	3.4–3.51	7.23	116	3.41–3.51		3.34–3.57	-	-	-
3″		7.42	120					-	-
4″		-	156					-	-
5″		7.42	120					-	-
6″	1.24–1.35	7.23	116	1.23	17.87	1.23	14	-	-
3″,4″,5″-OCH_3_	3.4–3.51	-	-	3.48–3.51	70	3.51 *s*	70	-	-
1’’’	3.4–3.51	3.65	59.84	3.41–3.51	66	3.34–3.57	67	3.17	49.15
2’’’	1.62–1.63	2.88 *s*	50.52	1.72–1.73	20–30	2.00	22–33	1.16–1.28	31.18
3‴	1.24–1.35	-	206	1.21–1.24		1.21			29
4‴		2.11 *s*	48.99						25
5‴		1.29 br.s	25.13–32.0						26
6‴									26
7‴								2.47	12.24–32.0
8‴								1.16–1.28	
9‴		1.21–1.23 *d*		1.76					
10‴		-	20.82	0.84	14.37			1.84	
11‴		-	-			1.24		0.83 *m*	
12‴	1.62–1.63	-	-	-	-	0.83	11	0.89 *m*	
13‴	0.86–0.90	-	-	-	-			2.47 *s*	
14‴		-	-	-	-	-	-	2.36 *d*	
15‴	-	-	-		-	-	-	2.36 *d*	

**Table 2:** Tentatively identified fatty acids in aerial parts of the petroleum ether extract *A. canariense* L.

Retention Time	Fatty Acid	Type	Percentage
20.8	Pentadecanoic acid (C15:0)	Saturated	1.91%
21.12	Cis-10-Pentadecenoic acid (C15:1)	Unsaturated	0.55%
23.936	Heptadecanoic acid (margaric acid, C17)	Saturated	0.72%
37.24	Cis-11-Eicosenoic acid (gondoic acid, C20:1)	Unsaturated	7.39%
43.20	Docosanoic acid (Behenic acid, C22:0)	Saturated	28.30%
47.01	Tricosanoic acid (Tricosylic acid, C23:0)	Saturated	43.08%
49.14	Tetracosanoic (Lignoceric acid, C24:0)	Saturated	0.76%
52.03	Nervonic acid (C24:1)	Unsaturated	8.94%
Saturated fatty acids			74.8%
Unsaturated fatty acids			16.9%

**Table 3:** Cytotoxic and anticholinestrase activities of the *A. canariense* L. extracts.

Sample	AChE Inhibitory Effect IC_50 (ng/mL)_	HCT-116 IC_50_ µg/mL	MCF-7 IC_50_ µg/mL	HepG-2 IC_50_ µg/mL
Alkaloid fraction	183.43 ± 38.98	14.40 ± 0.8	28.00 ± 1.2	21.00 ± 0.4
Aqueous alkaloid fraction	139.27 ± 21.40			
Methanolic extract	112.24 ± 7.73	21.20 ± 0.6	40.50 ± 3.1	26.40 ± 0.3
Dichloromethane	62.48 ± 1.31			
Tacrine	27.29 ± 0.49			
Doxorubicin		0.23 ± 0.17	0.42 ± 0.35	0.46 ± 0.2

**Table 4:** Mean inhibition zones in mm of alkaloidal and methanolic *Aizoon canariense* L. extracts.

Tested M.O	*Aizoon canariense* L. Alkaloid	*Aizoon canariense* L. MeOH	Control
**Fungi**			Ketoconazole
*Aspergillus flavus* (RCMB 002002)	NA	16 ± 1.5 ***	16 ± 1.5 ***
*Candida albicans* (RCMB 005003, ATCC)	16 ± 2 **	12 ± 1.0 **	20 ± 1.5 ***
**Gram-Positive Bacteria**			Gentamycin
*Staphylococcus aureus* (RCMB 010010)	13 ± 1.5 **	15 ± 1.0 **	24 ± 2.0 ***
*Bacillus subtilis* (RCMB 015, NRRL B-543)	14 ± 2 **	13 ± 1.5 **	26 ± 2 ***
**Gram-Negative Bacteria**			Gentamycin
*Salmonella typhimurium* (RCMB 006, ATCC 14028)	15 ± 2.0 **	13 ± 1.5 **	17 ± 1.5 ***
*Escherichia coli* (RCMB 010052, ATCC25955)	16 ± 1.5 ***	18 ± 2.0 ***	30 ± 2.0 ***

NA: No activity. Values are expressed as means of triplicate determination (n = 3) ± standard deviation. The statistical significance of the results was tested using one-way analysis of variance (ANOVA) and Tukey–Kramer multiple comparisons test. The *p* value significance was represented as an asterisk (**) for *p* < 0.01 and three asterisks (***) for *p* < 0.001.

**Table 5:** Minimum inhibitory concentration (MIC) values in μg/mL for alkaloid and methanolic *Aizoon canariense* L. extracts.

Tested Micro-Organism	Tested Extract		
	*A. canariense* L. Alkaloid	*A. canariense* L. MeOH	Standard
**FUNGI**			Amphotericin B
*Aspergillus flavus* (RCMB 002002)	NA	1250	0.98
*Candida albicans* (RCMB 005003, ATCC)	625	2500	0.49
**Gram-Positive Bacteria**			Ampicillin
*Staphylococcus aureus* (RCMB 010010)	1250	625	0.49
*Bacillus subtilis* (RCMB 015, NRRL B-543)	2500	625	0.49
**Gram-Negative Bacteria**			Gentamicin
*Salmonella typhimurium* (RCMB 006, ATCC 14028)	1250	2500	0.98
*Escherichia coli* (RCMB 010052, ATCC25955)	312.5	622	3.9

NA: No activity.

## Data Availability

Not available.

## Supplementary Materials

The following are available online. Figure S1: Mass spectra of compounds **1**, **2**, **3**, **4**, **5** positive mode and 5 negative mode; Figure S2: ^1^H-NMR of compound **1**; Figure S3: ^1^H-NMR of compound **2**; Figure S4: 1D APT ^13^CNMR of compound **2**; Figure S5: HSQC of compound **2**; Figure S6: COSY of compound **2**; Figure S7: ^1^H-NMR of compound **3**; Figure S8: ^13^C-NMR of compound **3**; Figure S9: HSQC of compound **3**; Figure S10: magnified HSQC of compound **3**; Figure S11: ^1^H-NMR of compound **4**; Figure S12: ^13^C-NMR of compound **4**; Figure S13: HSQC of compound **4**; Figure S14: ^1^H-NMR of compound **5**; Figure S15: HSQC of compound **5**.
