# Supplementary material for: New Adenosine Derivatives from Aizoon canariense L.: In Vitro Anticholinesterase, Antimicrobial, and Cytotoxic Evaluation of Its Extracts
Source: Molecules. 2021 Feb 24;26(5):1198. doi: 10.3390/molecules26051198 (PMC7956659; doi:10.3390/molecules26051198)
Supplement: Supplementary file 1 [file molecules-26-01198-s001.pdf]

Supplementary material

New Adenosine Derivatives from *Aizoon canariense* L.: *In Vitro* Anticholinesterase, Antimicrobial, and Cytotoxic evaluation of its extracts

Riham O. Bakr,[a], Mohammed F. El-Behairy [b], Ahmed M. Elissawy [c,d], Hanan Elimam [e,f], and Marwa A.A.Fayed*[g]

[a]*Department of Pharmacognosy, Faculty of Pharmacy, October University for Modern Sciences and Arts (MSA), Giza, Egypt.*

[b] *Department of Organic and Medicinal Chemistry, Faculty of Pharmacy, University of Sadat City, Sadat City 32897, Egypt*

[c]*Center for Drug Discovery Research and Development, Ain Shams University, Egypt*

[d]*Pharmacognosy Department, Faculty of Pharmacy, Ain Shams University, Cairo, Egypt.*

[e]*Department of Biochemistry, Faculty of Pharmacy, University of Sadat City, Sadat City, Egypt.*

[f] *Department of Medicine, McGill University Health Centre, National Research Institute, McGill University, Montreal, Quebec, Canada.*

[g*]*Department of Pharmacognosy, Faculty of Pharmacy, University of Sadat City, Sadat City 32897, Egypt.*

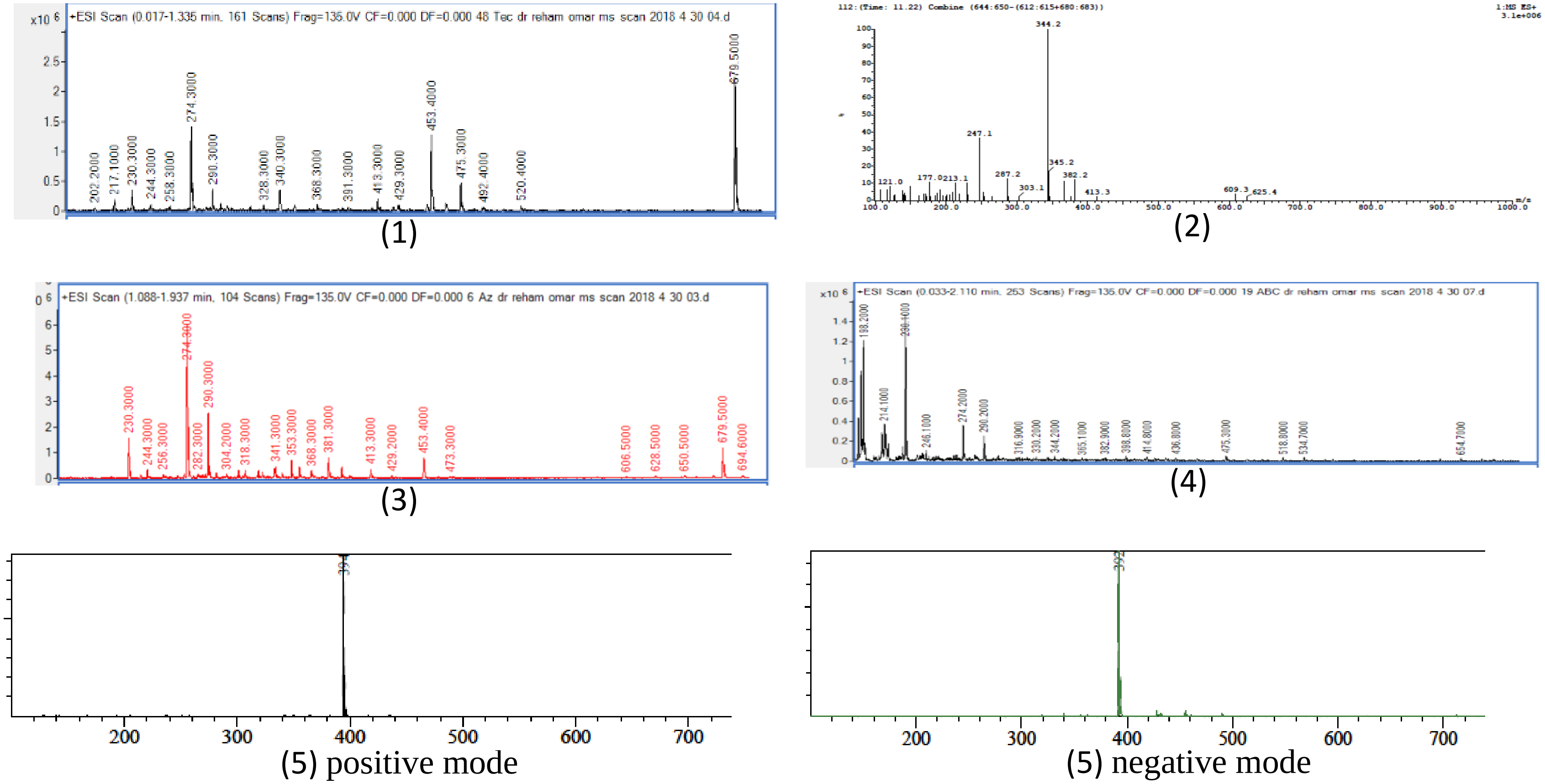

Figure S1: Mass spectra of compounds 1, 2, 3, 4, 5 positive mode and 5 negative mode.

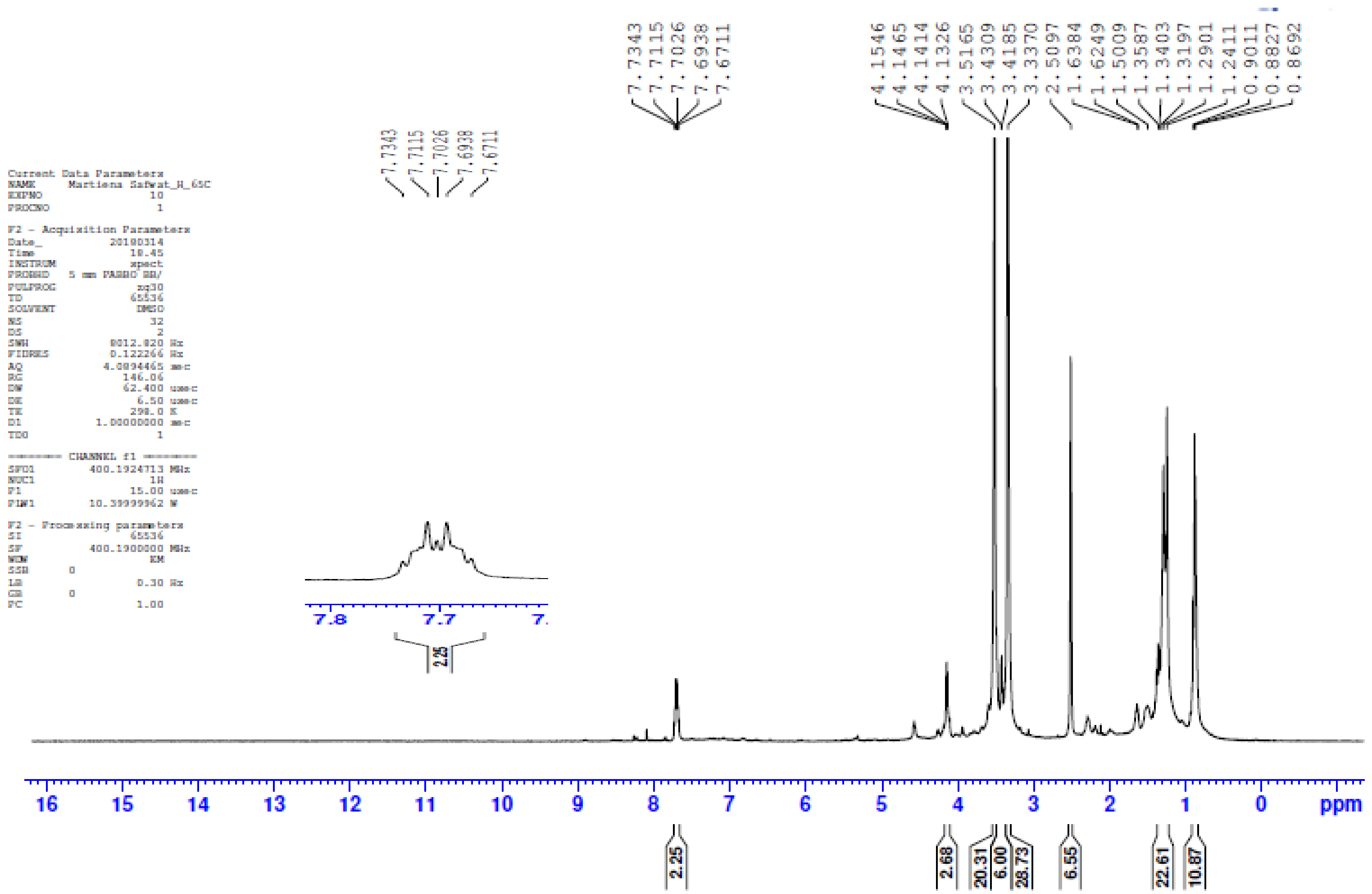

Figure S2: ^{1}HNMR of compound 1

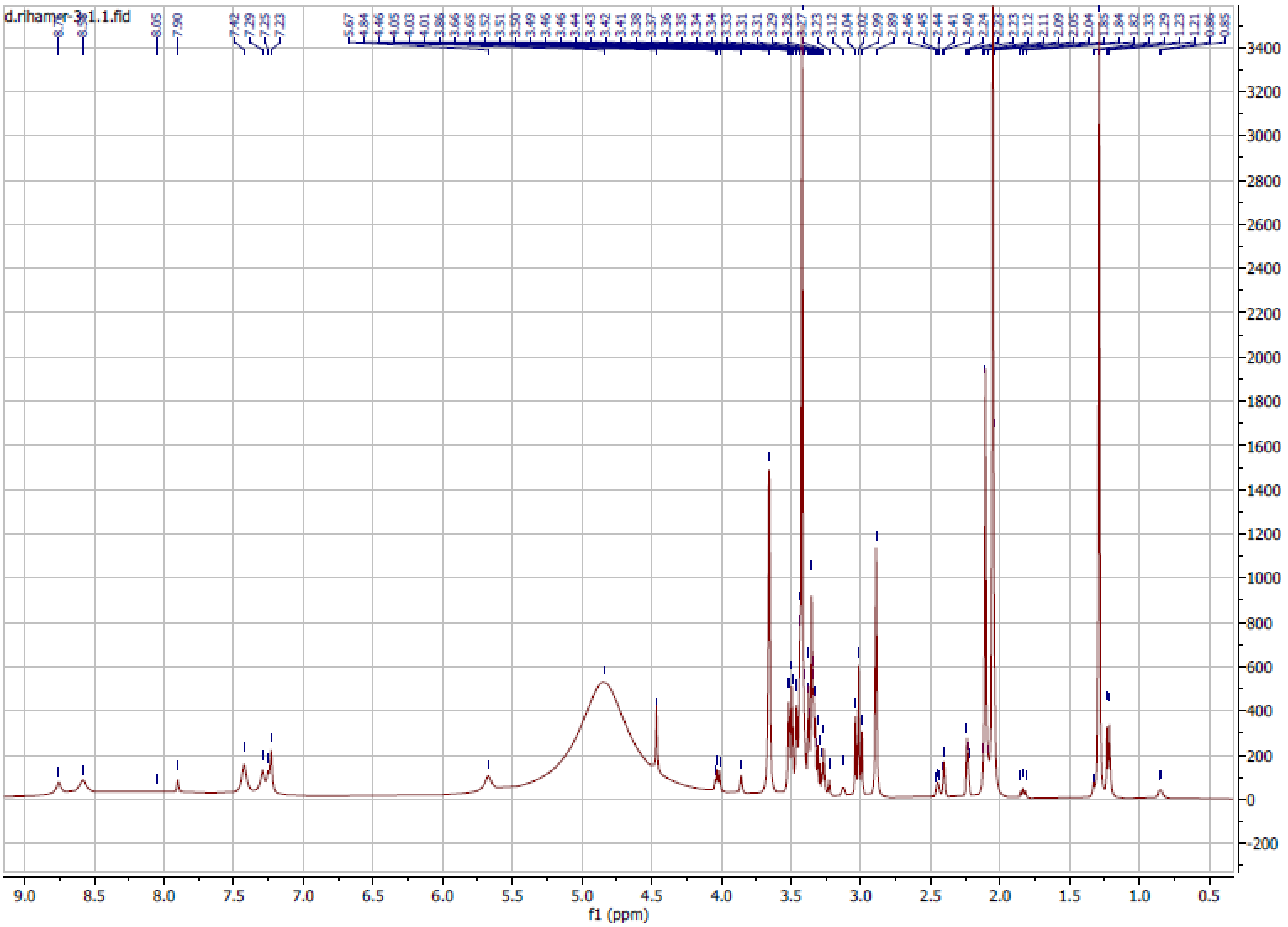

Figure S3: ^{1}HNMR of compound 2

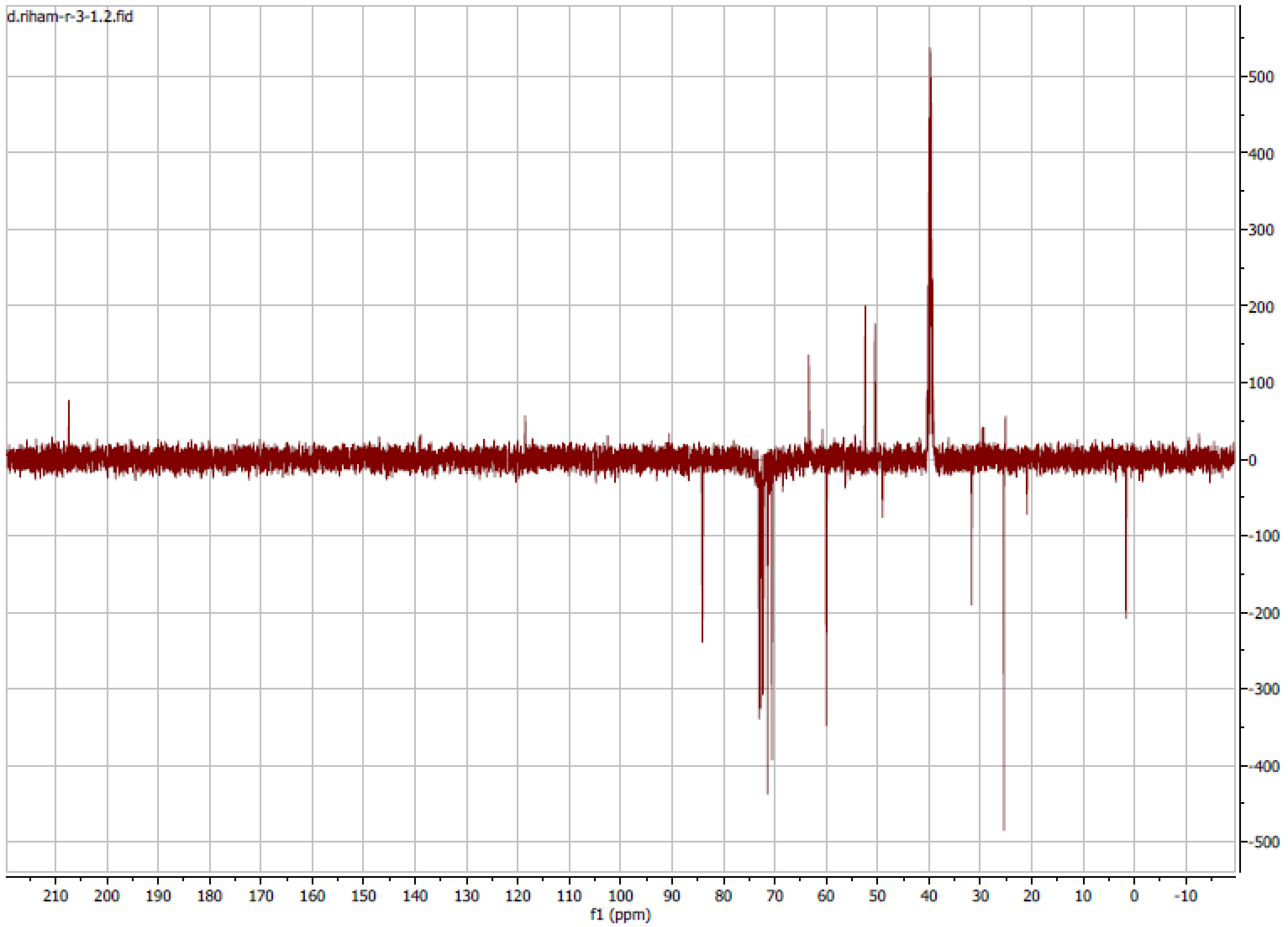

Figure S4: 1D APT ^{13}CNMR of compound 2

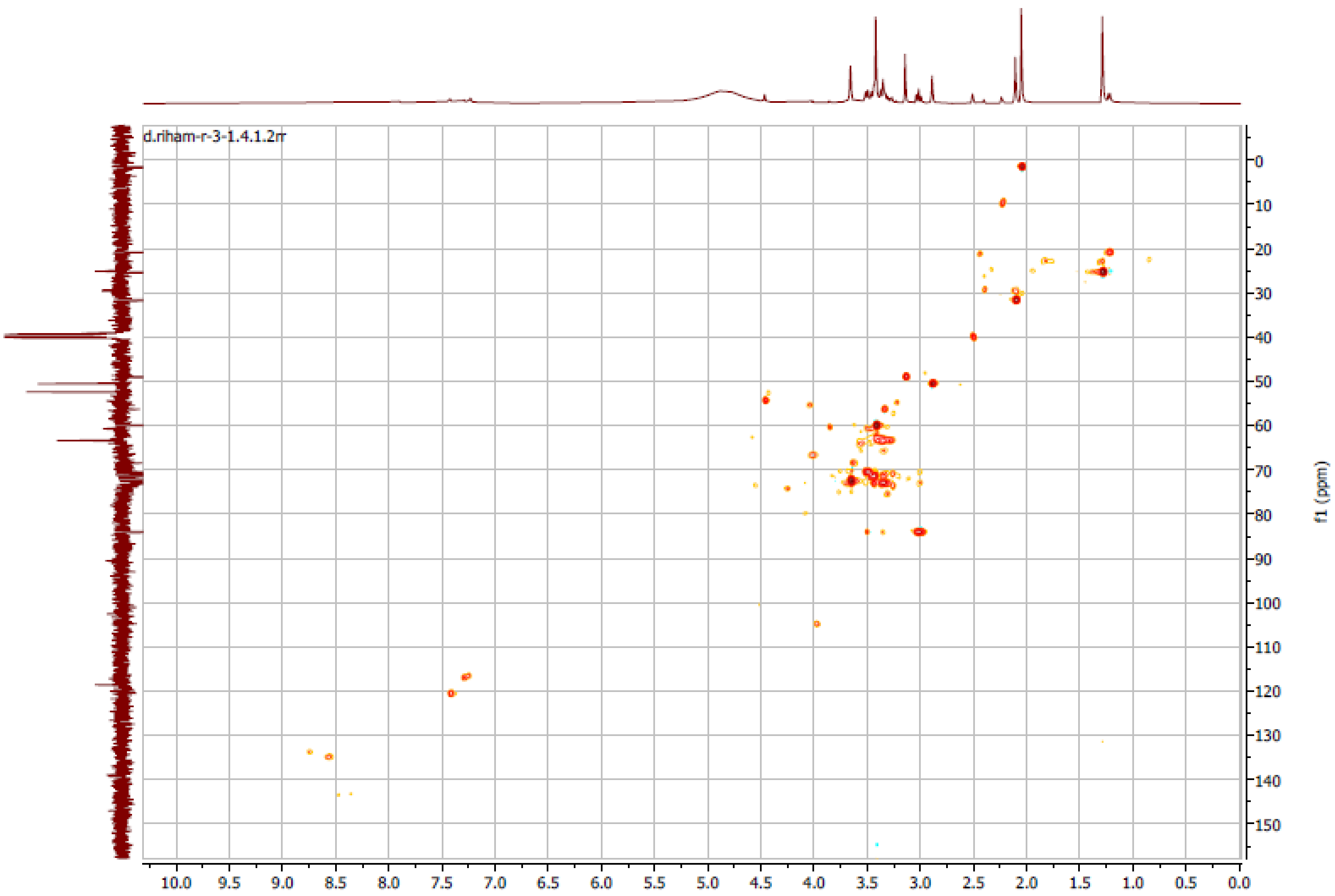

Figure S5: HSQC of compound 2

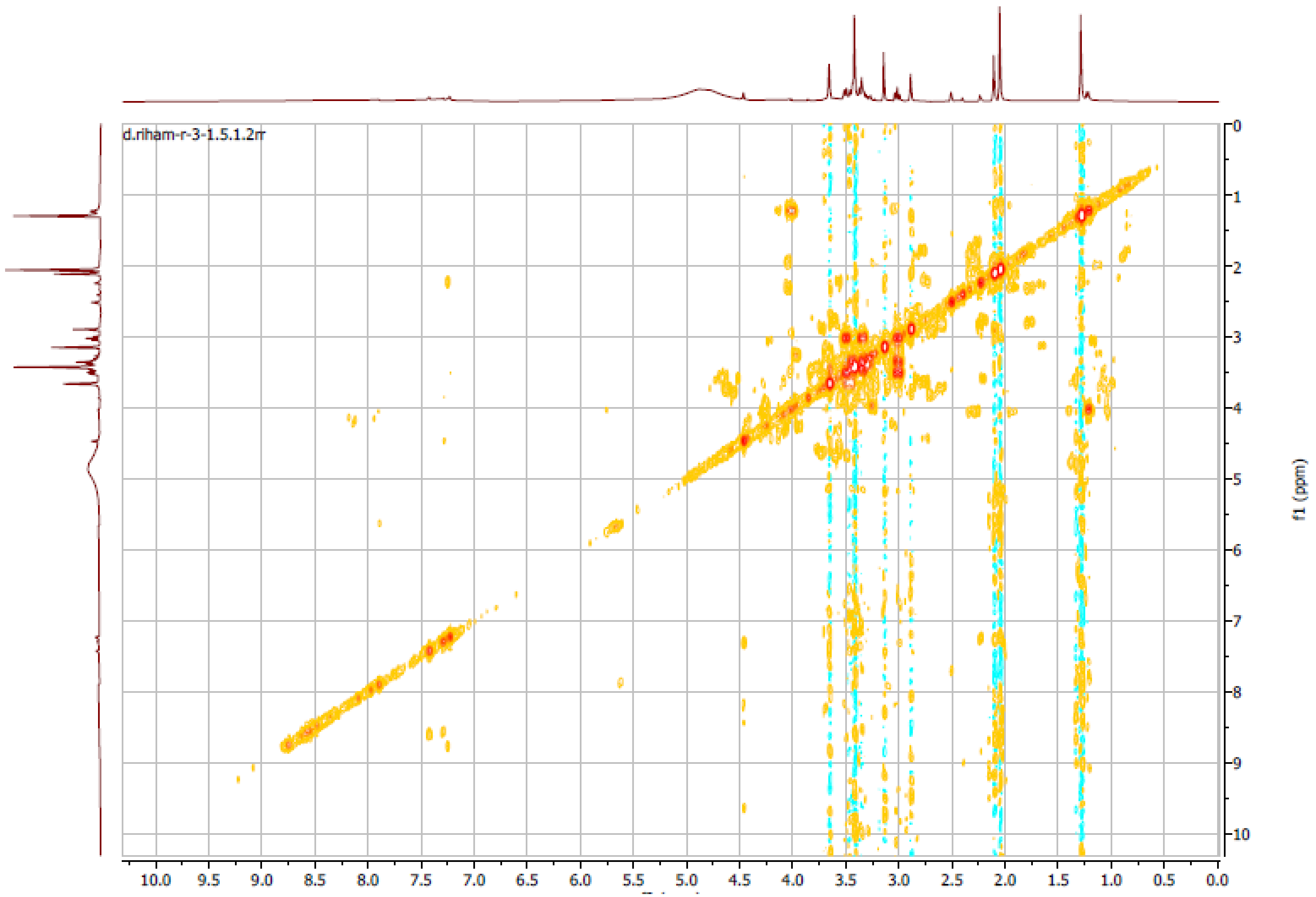

Figure S6: COSY of compound 2

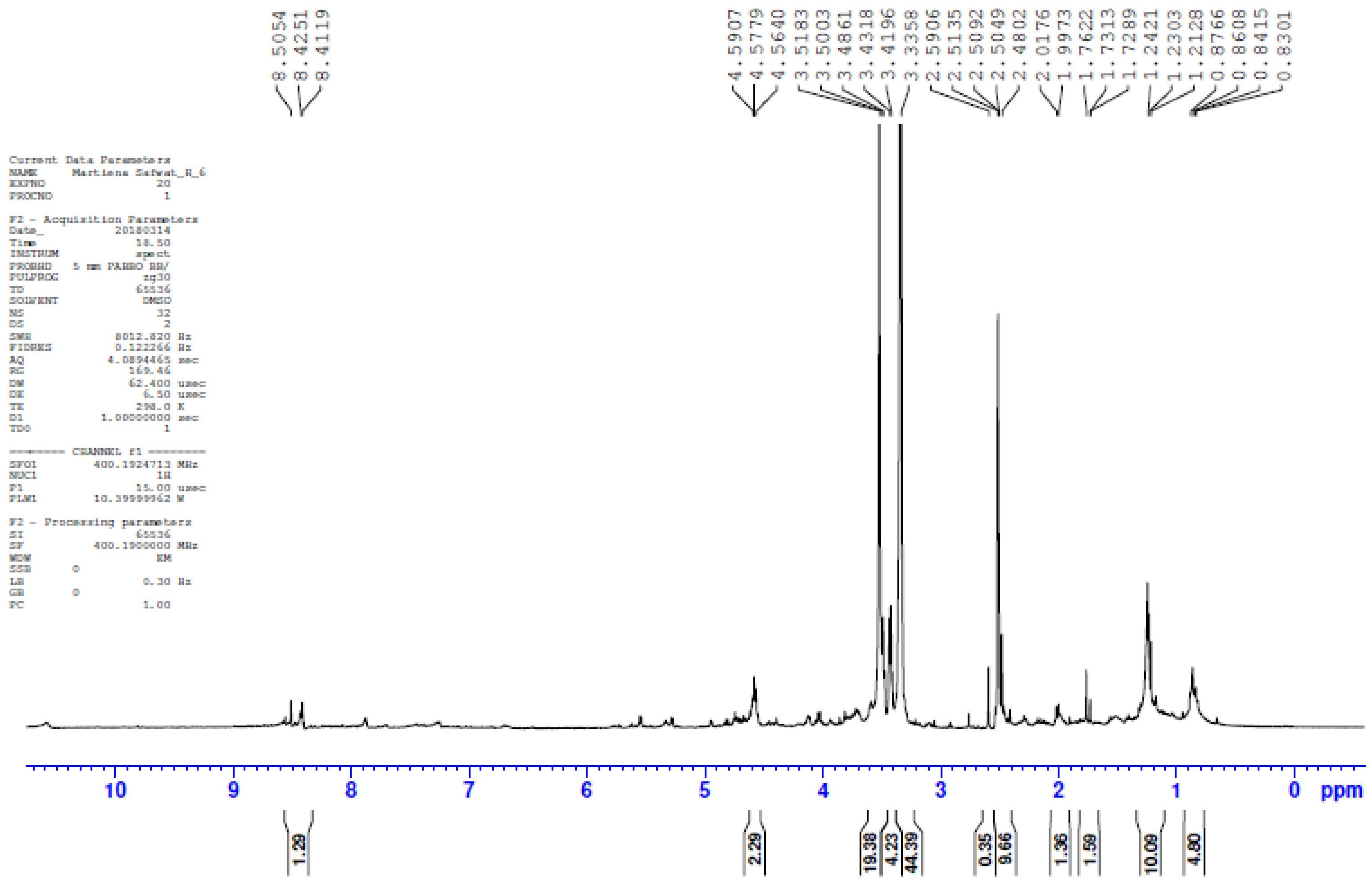

Figure S7: ^{1}HNMR of compound 3

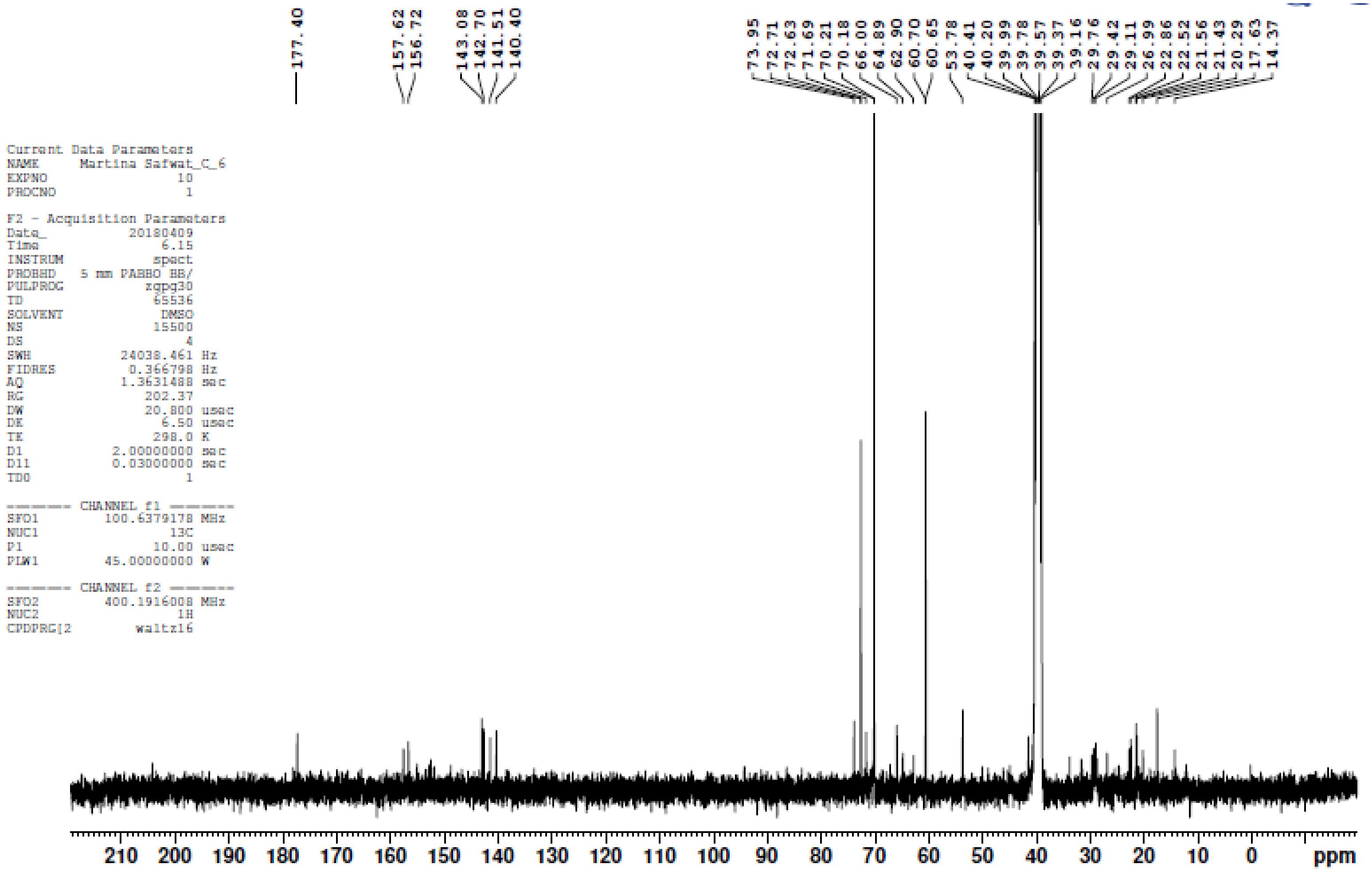

Figure S8: ^{13}CNMR of compound 3

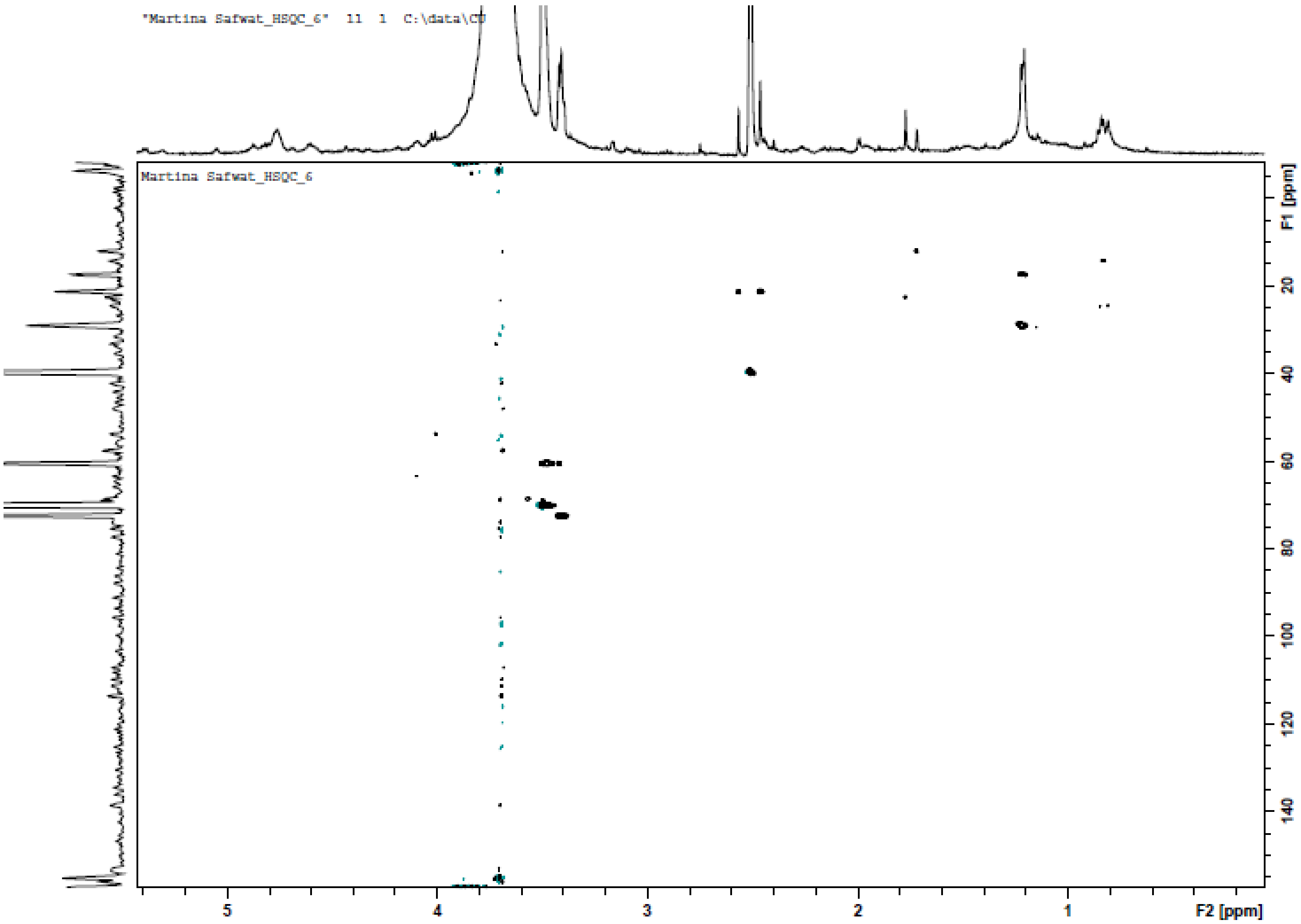

Figure S9: HSQC of compound 3

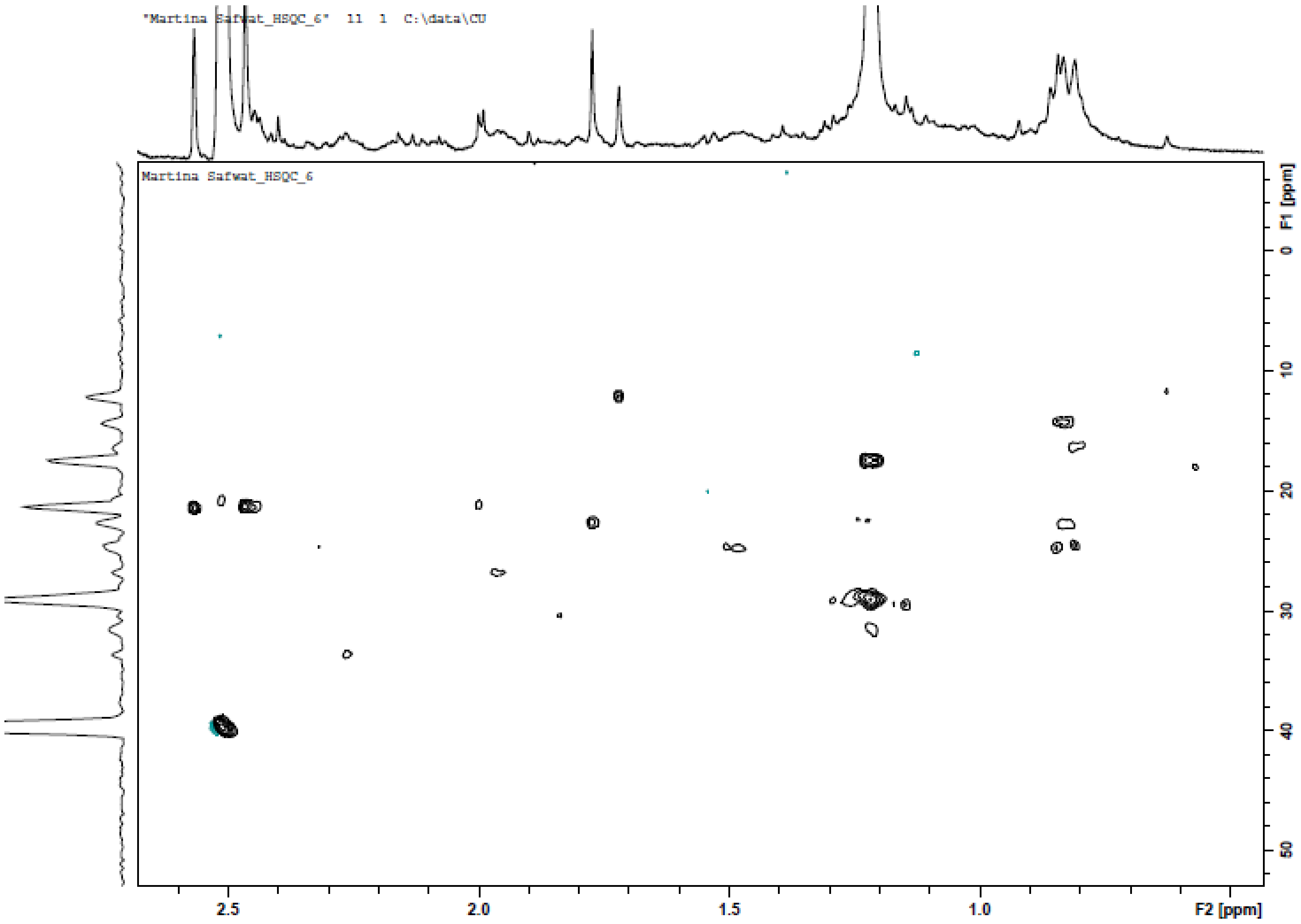

Figure S10: magnified HSQC of compound 3

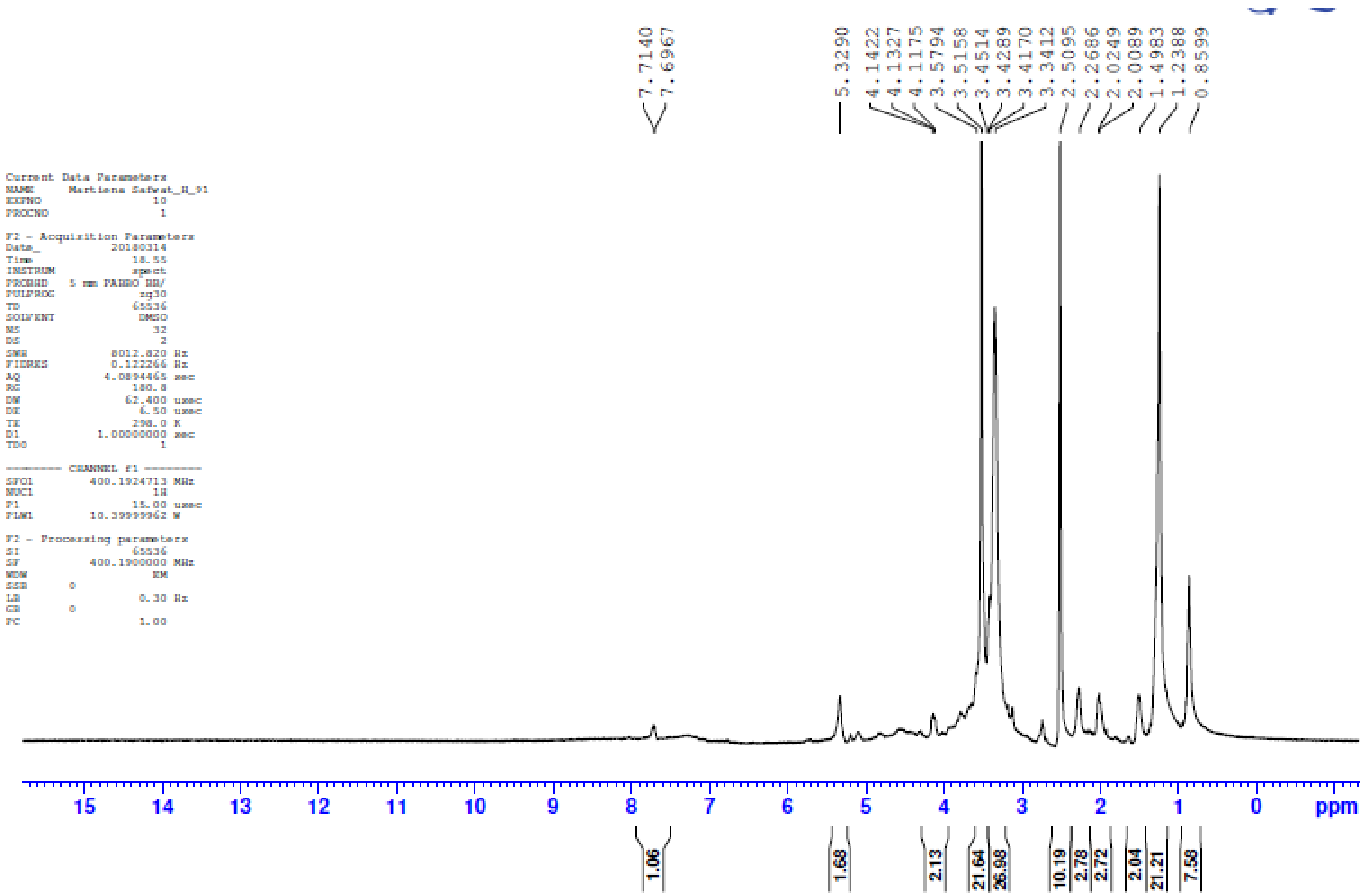

Figure S11: ^{1}HNMR of compound 4

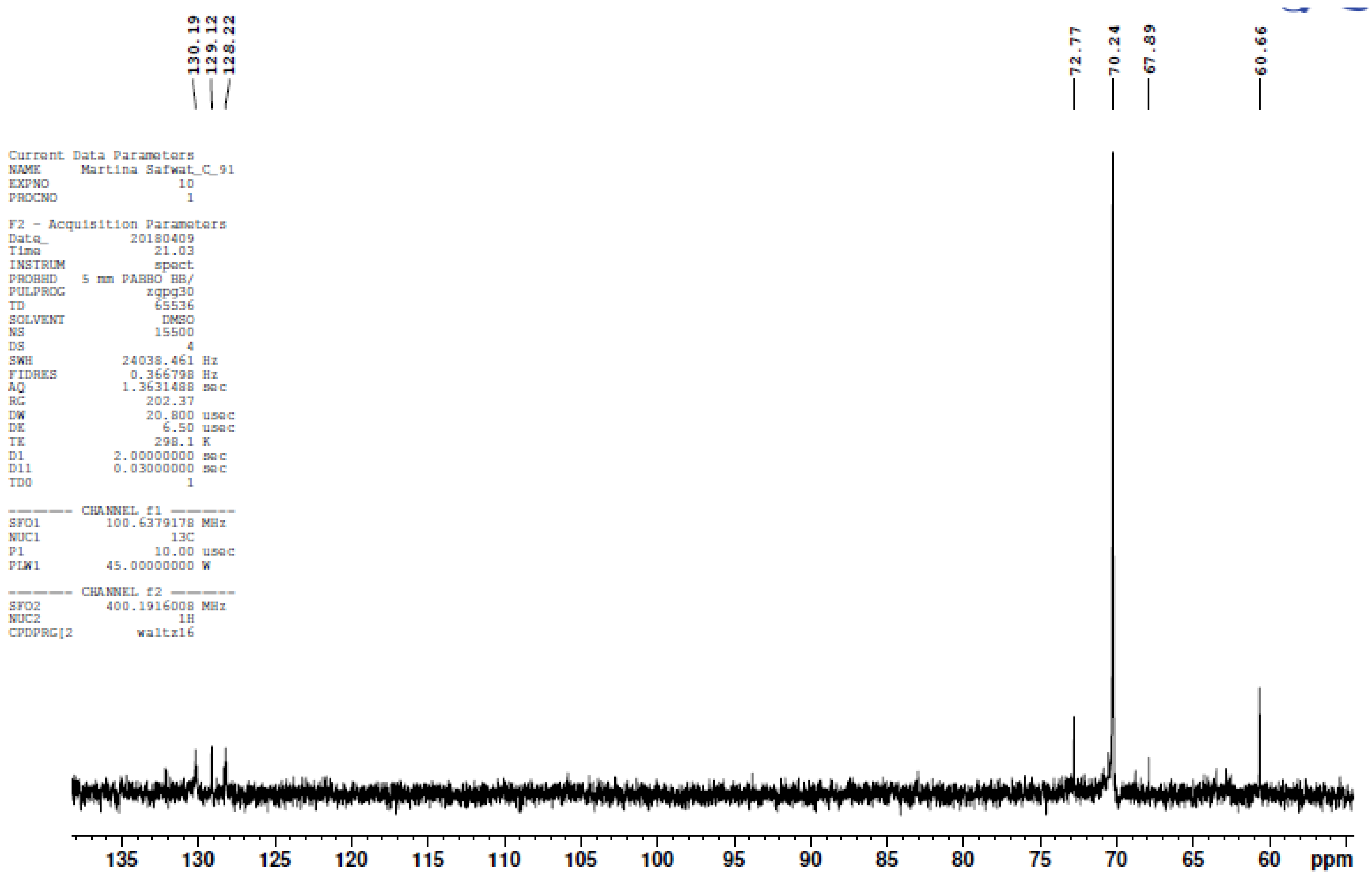

Figure S12: ^{13}CNMR of compound 4

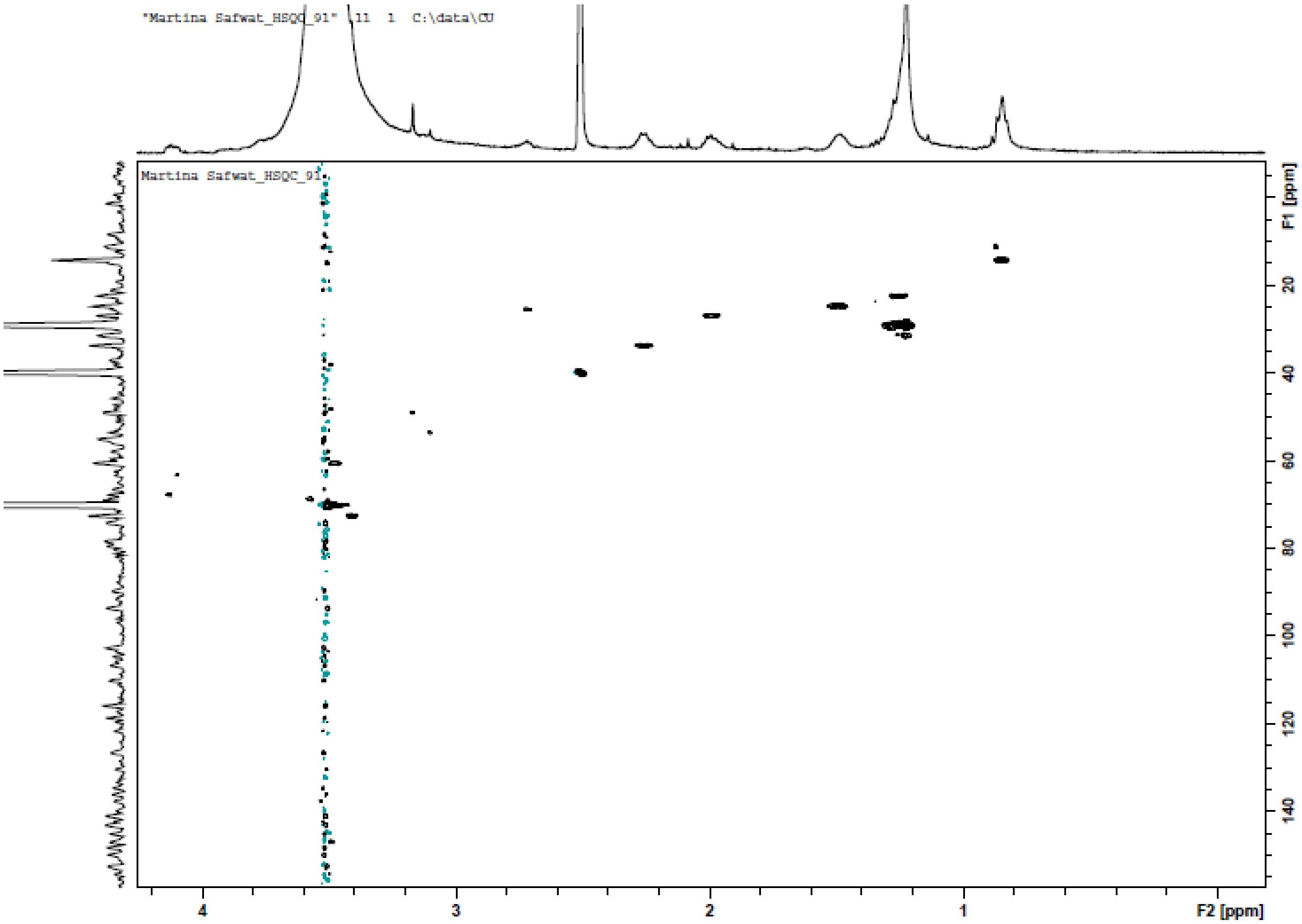

Figure S13: HSQC of compound 4

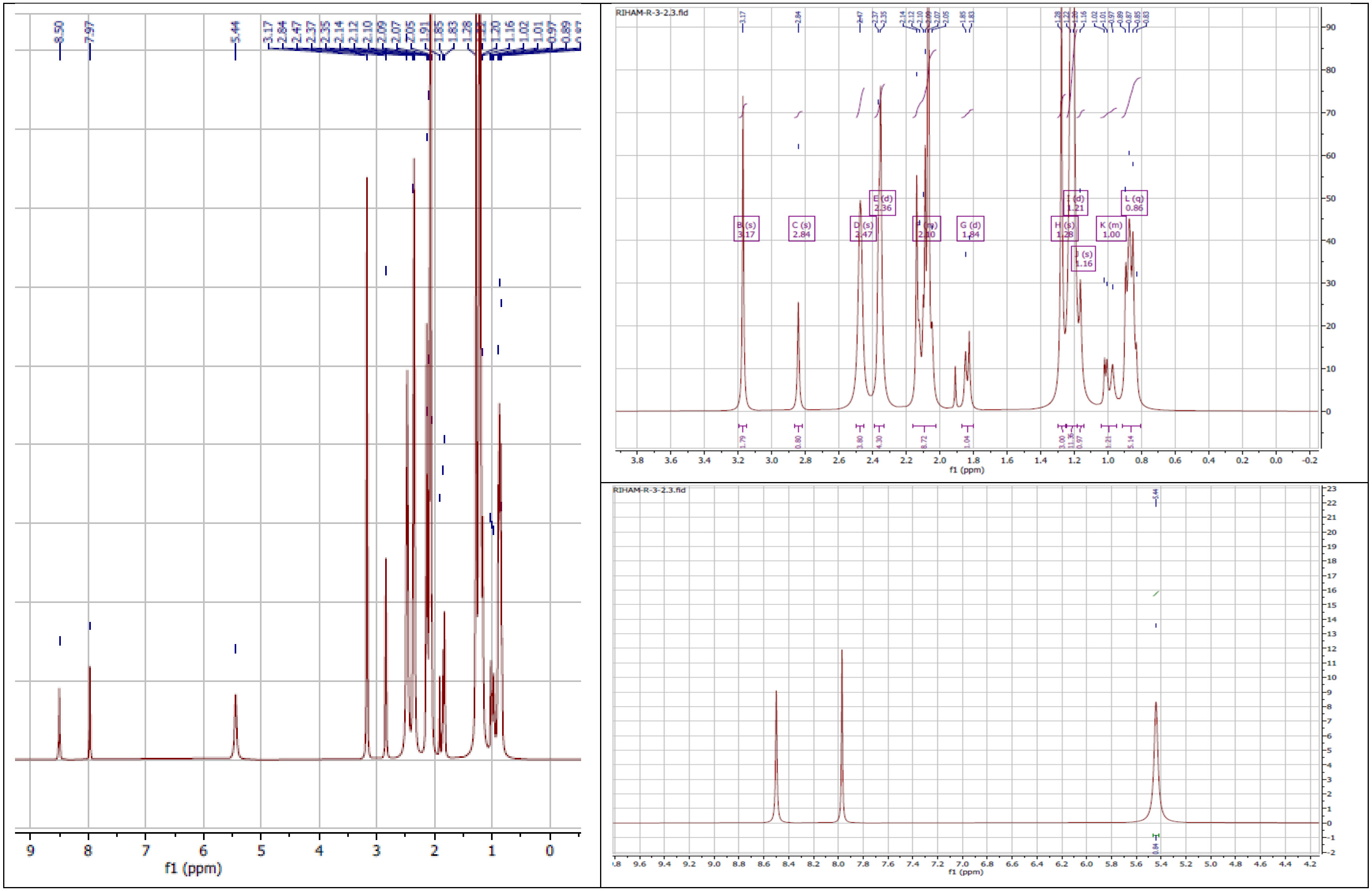

Figure S14: ^{1}HNMR of compound 5

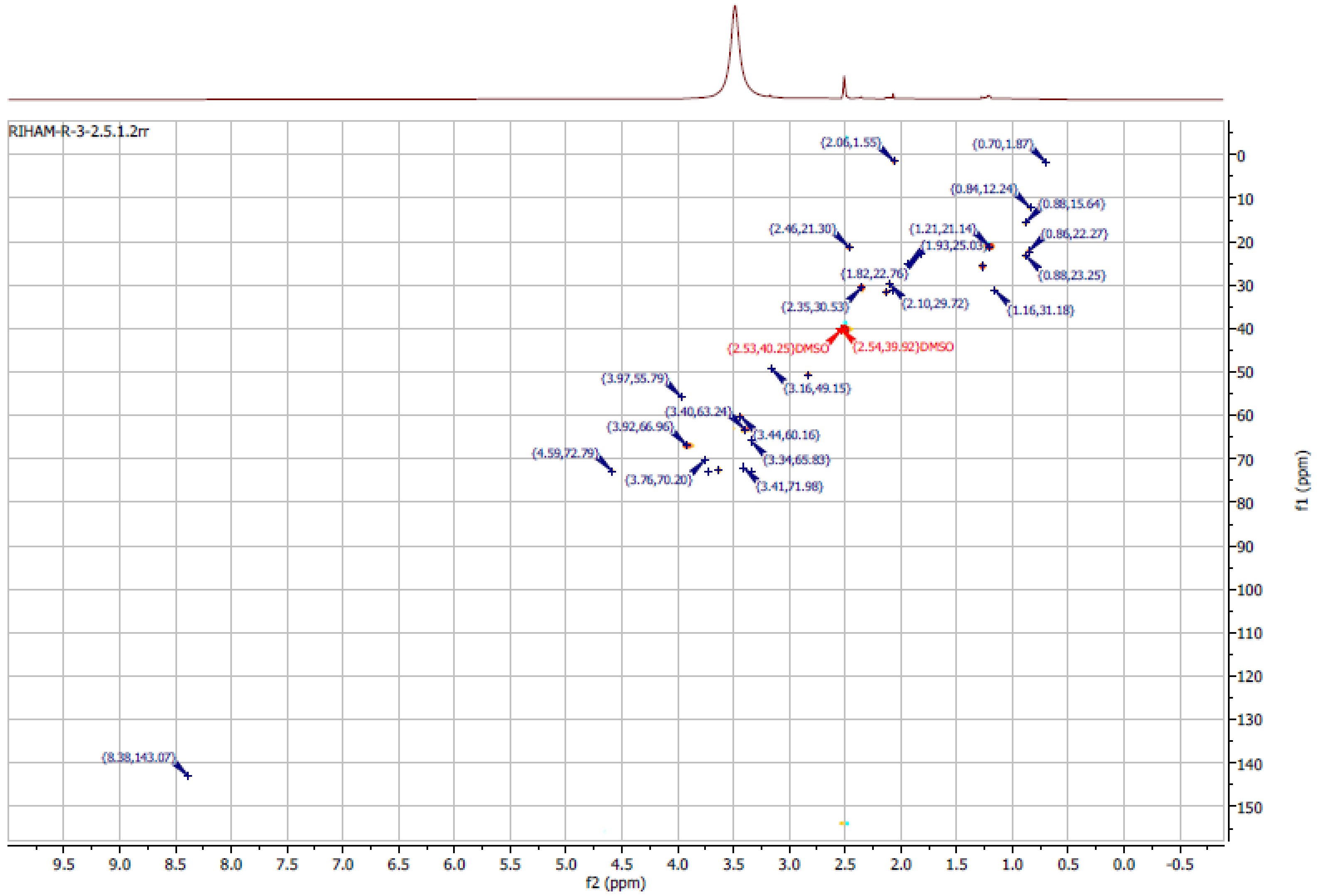

Figure S15: HSQC of compound 5

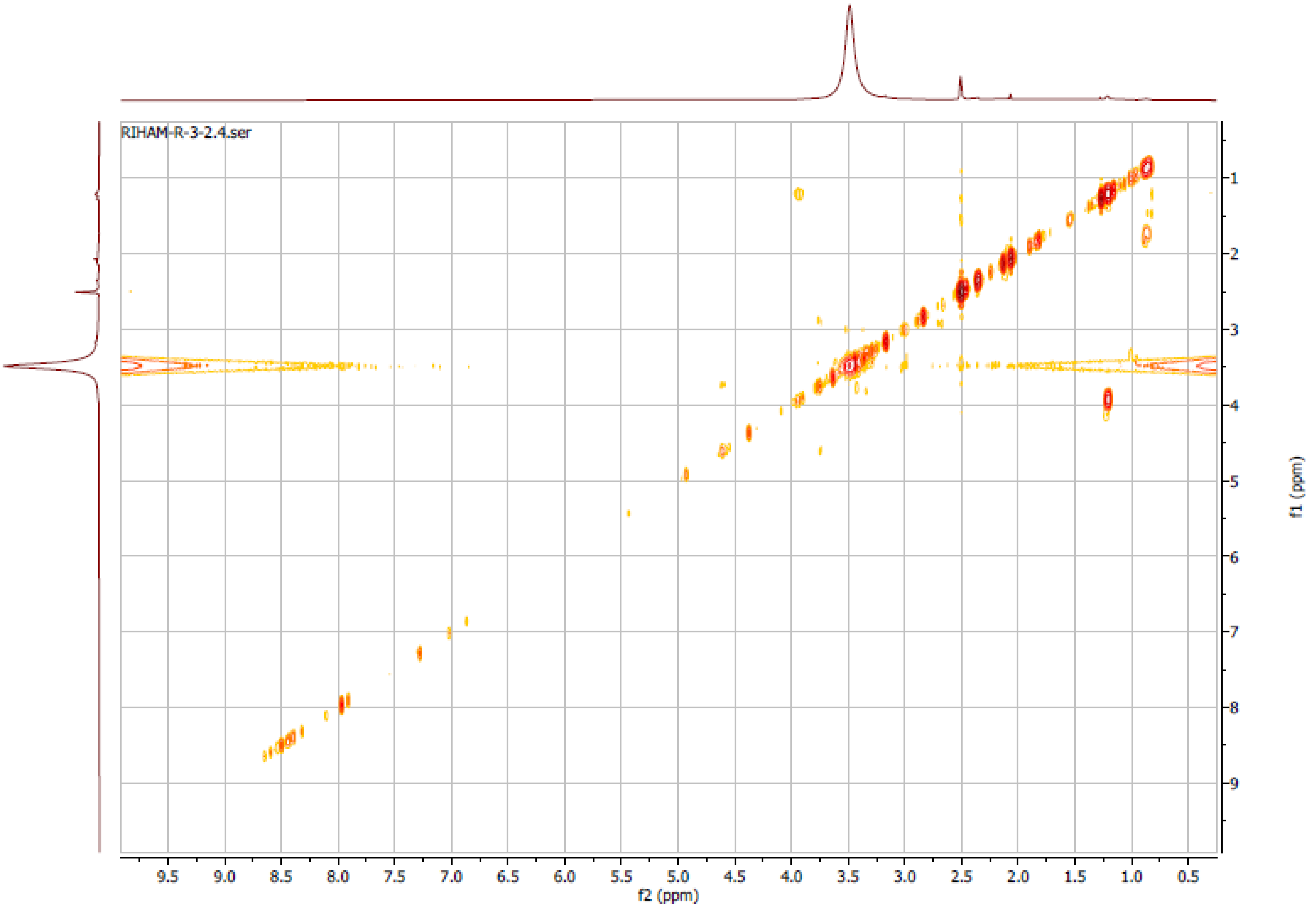

Figure S16: COSY of compound 5

Table S1: Anticholinesterase raw data

	100 ug/ml												1000 ug/ml										
	Methanolic Extract	Dichloromethane fraction	Alkaloid Fraction	Aqueous Alkaloid Fraction	Tacrine		Methanolic Extract	Dichloromethane fraction	Alkaloid Fraction	Aqueous Alkaloid Fraction	Tacrine		Methanolic Extract	Dichloromethane fraction	Alkaloid Fraction	Aqueous Alkaloid Fraction	Tacrine		Methanolic Extract	Dichloromethane fraction	Alkaloid Fraction	Aqueous Alkaloid Fraction	Tacrine
**R1**	0.80448	0.5952	0.77426	1.16641	5.91511	**R1**	18.5716	39.5542	15.9674	14.4284	45.2169	**R1**	50.6554	60.4039	44.1026	49.4663	67.1802	**R1**	74.4113	72.2995	68.7809	71.5929	89.2919
**R2**	0.85448	0.6652	0.88426	1.3641	6.1951	**R2**	20.5816	33.5442	14.749	17.5284	43.2169	**R2**	55.6754	62.0359	49.0216	46.4963	69.1802	**R2**	73.4113	74.2015	70.7809	73.5929	90.2919
**R3**	0.82448	0.7952	0.67436	1.06641	4.3151	**R3**	16.5716	40.5521	17.4674	11.4294	42.2169	**R3**	51.6554	58.4039	40.2306	52.4663	63.1102	**R3**	75.4013	70.2995	66.7309	65.5929	85.2919
**mean**	0.827813333	0.6852	0.777626667	1.198973333	5.4751	**mean**	18.57493	37.8835	16.06127	14.46206667	43.55023	**mean**	52.66207	60.28123	44.4516	49.4763	66.4902	**mean**	74.40797	72.26683	68.76423	70.25956667	88.2919
**std**	0.025166115	0.101488916	0.104990492	0.151492944	1.014297787	**std**	2.005002	3.790923	1.361629	3.049639378	1.527525	**std**	2.657091	1.819105	4.405879	2.985012563	3.093267	**std**	0.995004	1.951205	2.025051	4.163331999	2.645751
